# Trend analysis of multi-level determinants of maternal and newborn postnatal care utilization in Pakistan from 2006 to 2018: Evidence from Pakistan Demographic and Health Surveys

**DOI:** 10.1186/s12889-023-15286-7

**Published:** 2023-04-04

**Authors:** Sarosh Iqbal, Sidra Maqsood, Rubeena Zakar, Florian Fischer

**Affiliations:** 1 Department of Sociology, School of Social Sciences & Humanities, University of Management & Technology, Lahore, Pakistan; 2 Department of Sociology, Government College University, Lahore, Pakistan; 3 Department of Public Health, Institute of Social & Cultural Studies, University of the Punjab, Lahore, Pakistan; 4 Institute of Public Health, Charité – Universitätsmedizin Berlin, Berlin, Germany

**Keywords:** Postnatal care, Maternal PNC, Newborn PNC, Pakistan

## Abstract

**Background:**

Postnatal care (PNC) is crucial for maternal and newborn health. Healthcare-seeking practices within the postpartum period help healthcare providers in early detection of complications related to childbirth and post-delivery period. This study aims to investigate trends of PNC utilization from 2006 to 2018, and to explore the effects of multi-level determinants of both maternal and newborn PNC in Pakistan.

**Methods:**

Secondary data analysis of the last three waves of the nationally representative Pakistan Demographic and Health Surveys (PDHSs) was conducted Analysis was limited to all those women who had delivered a child during the last 5 years preceding each wave of PDHS Bivariate and multivariate logistic regression was applied to determine the association of maternal and newborn PNC utilization with multi-level determinants at individual, community, and institutional levels.

**Results:**

In Pakistan, an upward linear trend in maternal PNC utilization was found, with an increase from 43.5 to 63.6% from 2006 to 2018. However, a non-linear trend was observed in newborn PNC utilization, with an upsurge from 20.6 to 50.5% from 2006 to 2013, nonetheless a decrease of 30.7% in 2018. Furthermore, the results highlighted that the likelihood of maternal and newborn PNC utilization was higher amongst older age women, who completed some years of schooling, were employed, had decision-making and emotional autonomy, had caesarean sections, and delivered at health facilities by skilled birth attendants. Multivariate analysis also revealed higher odds for women of older age, who had decision-making and emotional autonomy, and had caesarean section deliveries over the period of 2006–2018 for both maternal and newborn PNC utilization. Further, higher odds for maternal PNC utilization were found with parity and size of newborn, while less for ANC attendance and available means of transportation. Furthermore, increased odds were recorded for newborn PNC utilization with the number of children, ANC attendance, gender of child and mass media exposure from 2006 to 18.

**Conclusion:**

A difference in maternal and newborn PNC utilization was found in Pakistan, attributed to multiple individual (socio-demographic and obstetrics), community, and institutional level determinants. Overall, findings suggest the need to promote the benefits of PNC for early diagnosis of postpartum complications and to plan effective public health interventions to enhance women’s access to healthcare facilities and skilled birth assistance to save mothers’ and newborns’ lives.

## Background

Globally, approximately 810 women die every on average each day due to preventable causes related to pregnancy and childbirth [1]. The Maternal Mortality Ratio (MMR) is higher for the Asian region and even highest for the South Asian region, where almost one-fifth of the global maternal deaths occur. Similarly, neonatal deaths are also higher in Central and South Asia with 24 deaths per 1,000 live births [1].

The latest Pakistan Maternal Mortality Survey (2019) highlighted that although MMR has reduced from 276 deaths (as per 2006-07) to 186 deaths per 100,000 live births in the country, however, Pregnancy-Related Mortality Ratio (PRMR) is still higher with 255 deaths per 100,000 live births [2]. Further, the Newborn Mortality Rate (NMR) has also slowly reduced from 54 (as of 2006-07) to 41 deaths per 1,000 live births and the stillbirth rate to 31 pregnancy losses per 1,000 births in Pakistan [3]. Overall, statistics showed improvement over the period of time from 2006 to 07 to onwards, nonetheless, these are quite alarming, depicting that Pakistan is still lagging behind to achieve the Sustainable Development Goals (SDGs), ending preventable maternal and newborn deaths [4]. According to United Nations (UN), there is a dire need to address the issue of maternal and neonatal mortality as a top priority, reducing MMR to less than 70 deaths per 100,000 live births [2], and NMR to 12 deaths per 1,000 live births by 2030 [4].

A large strand of literature suggests preventing maternal and neonatal mortality, emphasizing skilled birth attendance, postnatal care facilities, provision, and access to modern contraception [4–7]. Evidence shows that most of the complications related to childbirth such as postpartum hemorrhage and various infections start instantly after birth, which put mothers’ and newborns’ health and lives at risk. These complications can be addressed by giving timely care to mothers and children in the period of postnatal care [7–12].

Postnatal care (PNC) is defined as care given during the first six weeks to the mother and her newborn, immediately after the birth of the placenta [13]. Literature suggests that the first 42 days after birth are very crucial, as the majority of maternal and neonatal deaths befalls during this period [14, 15]. Healthcare-seeking practices within the postpartum period help healthcare providers in early detection of complications related to childbirth and post-delivery period, enabling them to provide timely treatment to both mother and child to avoid any morbidity or mortality [14, 16]. In addition to saving mother and child from complications, PNC also provides opportunities for the new mothers to discuss their health-related issues, such as breastfeeding, required balanced nutrition, taking care of the child, and family planning [17].

Regardless of the importance of PNC for maternal and neonatal health, evidence revealed that most mothers and newborns do not obtain PNC from a skilled healthcare professional in developing countries [18, 19]. According to Pakistan Demographic Health Survey (PDHS) 2017-18, 36.4% of women and 69.3% of newborns did not receive PNC or a checkup by a health professional within six weeks after childbirth [20]. Further, there is also a disparity in terms of urban and rural areas of residence [20].

Pakistan, being a member state of the UN and signatory of SDGs, is committed to reducing maternal and newborn mortality rates to meet the UN target and address the issues of MMR and NMR. Predominantly, the SDGs are unmet due to maternal and neonatal deaths, and low-skilled birth assistance [21]. Therefore, it is imperative to provide skilled PNC to both mothers and newborns to ensure early diagnosis of complications for averting preventable maternal and neonatal deaths.

Given the context, there is limited research done on PNC in the country [22]. Although various studies have explored the maternal and newborn outcomes, particularly highlighting the significance of antenatal care and skilled birth assistance in Pakistan [23–25], there is a scarcity of research, examining trends of PNC for both mothers and newborns. Thus, bridging the gap in existing literature, this research article is aimed to examine trends of PNC utilization from 2006 to 2018 as well as explore the effects of multi-level determinants of both maternal and newborn PNC in Pakistan. This research is intended to unveil the trend of maternal and newborn PNC utilization from 2006 to 2018, examining the role of various underlying factors, contributing to and/or affecting the utilization of maternal and newborn PNC services at multi-levels, including individual (socio-demographics, obstetric), community and institutional levels.

### Conceptual framework

Seeking guidance from the literature [26–29], the conceptual framework of this research encompasses multi-level hierarchical factors, affecting maternal and newborn PNC utilization in local cultural settings of Pakistan. These factors are related to access to maternal and newborn healthcare services, and the healthcare-seeking behavior of the respondents, along with community-level determinants. This research has conceptualized that maternal and newborn PNC utilization is linked with three levels of determinants, i.e. individual-level factors, community-level, and institutional-level factors. A list of explanatory variables for each factor has been selected based on Andersen’s health-seeking behavioral model [30–34].

**Fig. 1:**
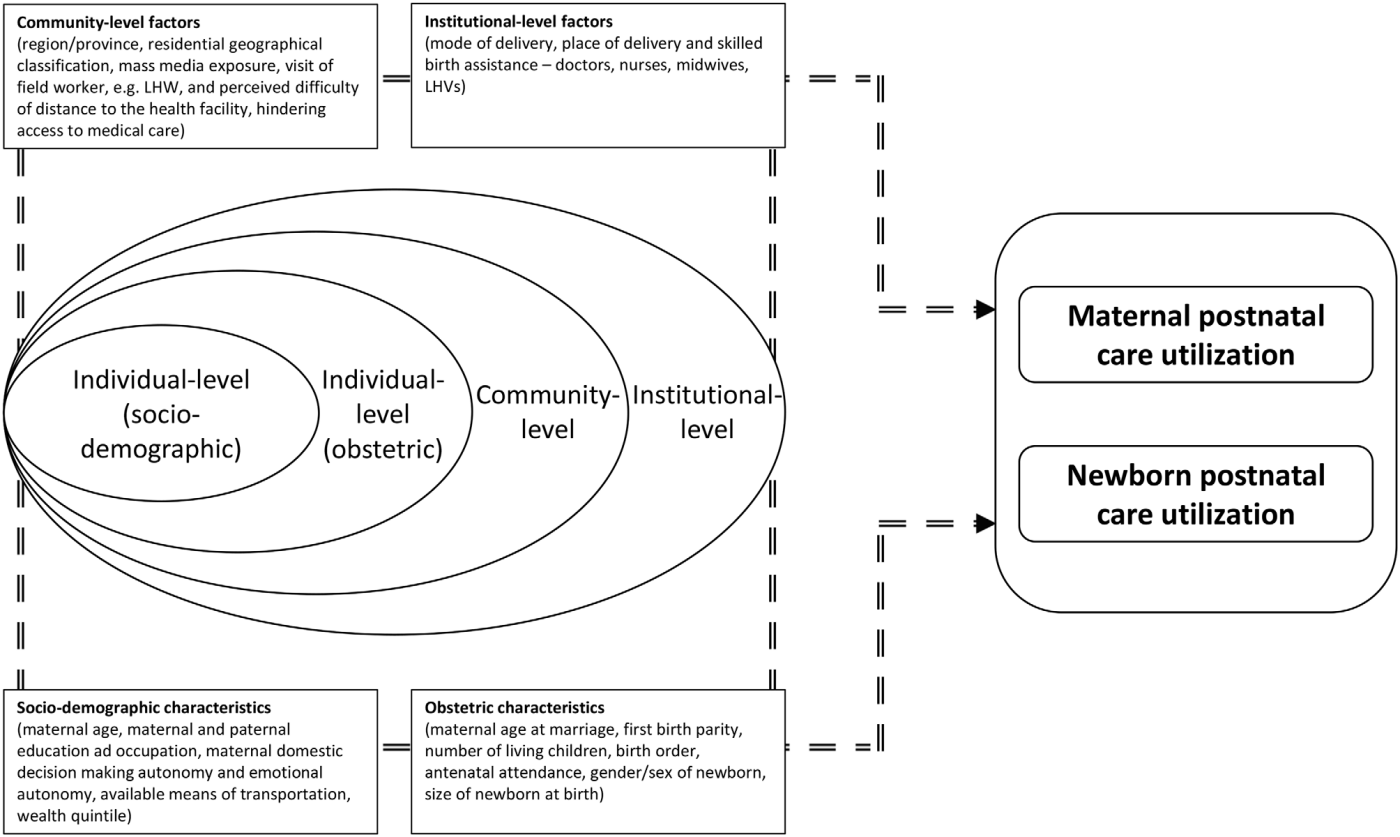
Hierarchical conceptual framework of maternal and newborn postnatal care utilization

The proposed conceptual framework accounts for individual women’s behavior, support from their families and community, as well as accessibility to maternal and newborn services in Pakistan. As illustrated in Fig. 1, the characteristics of the women and household factors (e.g., socio-demographic and obstetric) are considered as individual-level (level-1) predictors, whilst characteristics of the community are taken as group-level (level-2) predictors and facilities/institutional-level characteristics are grouped as level-3. This research conceptualizes that these three levels of predictors altogether contribute to maternal and newborn PNC utilization.

At an individual level, the socio-demographic characteristics included women’s age, women’s and husband’s educational status and occupation, women’s decision-making and emotional autonomy, available means of transportation, and wealth quintile. The obstetric characteristics comprised women’s age at marriage and first birth, parity, number of living children, birth order, antenatal attendance, sex/gender of newborn, and size of the newborn at birth.

At the community level, multiple factors have been considered, such as regions/provinces, residential geographical classification, mass media exposure, visit of a field worker, and perceived difficulty of distance to the health facility, hindering access to medical care.

Lastly, institutional level characteristics included mode of delivery, place of delivery, and skilled birth assistance.

## Methods

For this particular research, a secondary data analysis of the last three waves of the nationally representative and cross-sectional Pakistan Demographic and Health Surveys (PDHSs) was conducted. These include wave 2 (2006-07) [35], wave 3 (2012-13) [36] and wave 4 (2017-18) [37]. Since the data on PNC related variables was not collected during wave 1 (1990–1991) of PDHS [38], therefore, it is not part of the present study.

Under the international series of MEASURES DHS Program, the National Institute of Population Studies (NIPS) carried out PDHSs, about every five years to allow comparisons over time [35–37], with the exception from PDHS 1990-91. ICF International and the Pakistan Bureau of Statistics provided technical assistance for these surveys, whereas United States Agency for International Development provided financial assistance [35–37].

The PDHSs is a series of the largest representative and publicly available datasets at the household level, collected information of ever-married women of reproductive age between 15 and 49 years, including maternal and newborn PNC. Each of the PDHS waves applied a two-stage cluster random sampling design to interview married women of reproductive age (MWRAs) [35–37]. Primarily, urban and rural sampling units were selected, and secondly, eligible households with MWRAs were chosen for surveys [35–37]. During each wave of PDHS, data was collected by different field teams, each consisting of a supervisor, field editor, and four interviewers (1 male and 3 females). Further quality controllers, regional/provincial field coordinators, and core teams of NIPS and ICF supervised these field teams for quality assurance [35–37]. Concurrently with fieldwork, data processing, editing, and double data entry were also completed [35–37]. A set of questionnaires were used to collect data at community, household, and individual (women and men) levels during each wave of PDHS. This particular research used a standard women questionnaire for secondary data analysis, administered to MWRAs (aged 15–49 years), through a face-to-face method [35–37]. This women’s questionnaire also comprised of questions regarding obstetric care including maternal and newborn PNC [35–37]. Overall, the response rate for each wave of PDHS ranged from 93 to 94.5% [35–37].

**Fig. 2:**
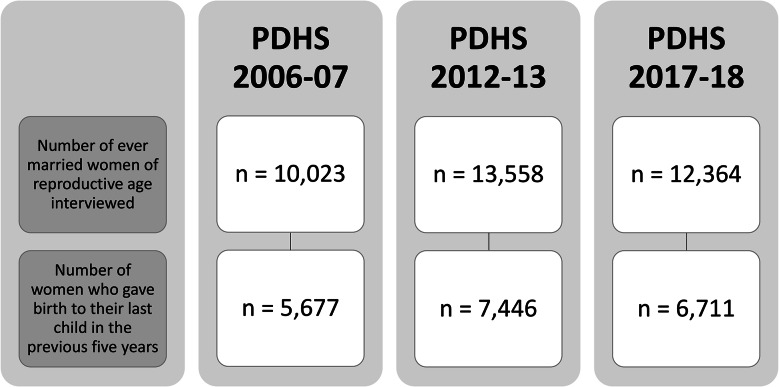
Sample selection with inclusion criteria

A sample of 10,023, 13,558 and 12,364 ever MWRAs were interviewed for PDHS 2006-07, 2012-13 and 2017-18 respectively [35–37]. However, this analysis is limited to all those women, who had delivered a child during the last 5 years preceding each wave of PDHS from 2006 to 2018, considering the research objectives. Thus, it yielded sample size 5,677, 7,446 and 6,711 women for PDHS 2006-07, 2012-13 and 2017-18 respectively [35–37], as shown in Fig. 2.

### Variables

#### Outcome variables

There are two outcome variables for this research, i.e. maternal PNC and newborn PNC utilization. Maternal PNC utilization represents those ever MWRAs who gave birth during the five years preceding each wave of the PDHSs and utilized PNC after childbirth. Maternal PNC utilization was derived from the following questions: “Anyone checked respondent before discharge/delivery?” and “Anyone checked respondent after discharge/delivery?” The women, who received PNC services were coded as ‘yes’, while those women, who didn’t receive PNC were coded as ‘no’.

Another outcome variable is newborn PNC utilization within the last 2 months, which was also coded into binary responses, i.e. ‘yes and no’.

#### Explanatory variables

Considering the datasets of PDHS, the multi-level explanatory variables were organized into three hierarchical levels. The characteristics of the women and household factors were considered as individual-level (level-1) predictors, whilst characteristics of the community were taken as group-level (level-2) predictors and facilities/institutional level characteristics were grouped as level-3. Following are the details of each level:

1. Individual-level characteristics.
  The individual level (women and household) characteristics include socio-demographic and obstetric-related variables. The socio-demographic characteristics include women’s age in years (15–24 years, 25–34 years, 35 years and above), women’s and husband’s educational status (no formal schooling, up to 5 years of schooling, 6–10 years of schooling, more than 10 years of schooling), women’s and husband’s occupation (not working/unemployed, professional/clerical/sales & services, agriculture, manual or household worker). The available means of transportation for the household were coded into the binary category, i.e. no vehicle or own vehicle. Here, own vehicle indicates the availability of a bicycle, motorcycle/scooter, or car/truck at the household level, capable to transport mothers. The composite index of household amenities was grouped into five wealth quintiles (poorest, poorer, middle, richer, and richest).
  Women’s domestic decision-making autonomy and emotional autonomy were also key predictors at the individual level. Women’s domestic decision-making autonomy measures the overall contribution of women in household decisions, such as spending one’s own and husbands’ earnings, making large household purchases, visiting family or relatives, and making decisions for healthcare. These were inferred from the following questions: i) “who (in your family) usually decides how to spend your earnings?”, ii) “who usually decides on making large household purchases?”, iii) “who usually decides on your visits to family or relatives?”, iv) “who usually decides on your healthcare?” and v) “who usually decides what to do with your husband/partner’s earnings?”. Possible responses to the first four questions were: respondent alone, husband/partner alone, respondent and husband/partner jointly, respondent and other person, someone else, family elders, or others. Nonetheless, for the last question regarding the decision of husband/partner’s earnings, an option of husband/partner does not bring in any money was also added along with other possible responses. For this research, all the responses to the above decision-making questions were dichotomized into one of two categories: whether the woman has ‘a say at all’ (either alone or jointly with the husband/partner or other person) was coded as ‘yes’ or whether she has ‘no say at all’ was coded as ‘no’ (in the case, when husband/partner, family elders or someone else makes the decision). This dichotomization of decision-making autonomy (yes/no) is consistent with the previous research work done using the DHS data [39, 40]. Based upon these five binary household decision-making indicator questions, a score of decision-making was computed for each woman, ranging from 0 to 5, where 0 showed ‘no say or autonomy’ and 1–5 reflected ‘say in any of the five household decisions or yes autonomy’. Cronbach’s alpha (α) for autonomy in domestic decision-making was found 0.88, 0.92, and 0.91 for PDHS 2006-07, 2012-13, and 2017-18 respectively, showing higher internal consistency.
  Women’s emotional autonomy was assessed, using their attitudes against women violence.
  Women’s agreement or disagreement represents their emotional autonomy [40]. It was inferred through the following five situations when sometimes a husband, being annoyed or angered, is justified in hitting or beating his wife: i) “if she goes out without telling husband?”, ii) “if she neglects the children?”, iii) “if she argues with husband?”, iv) “if she refuses to have sex with husband?” and v) “if she burns the food?”. Possible responses for each question were yes, no, and don’t know. For this research, yes and don’t know responses were given 0 scores, while no was scored as 1. Thus, a score ranged 0–5 was computed and dichotomized using the mean value. Thus, mothers, who had a score between 1 to 5 were categorized as ‘having emotional autonomy (disagree with wife-beating circumstances)’ and 0 scores indicated as ‘no emotional autonomy (agree with wife-beating situations). Cronbach Alpha (α) for emotional autonomy was found 0.913 and 0.908 for PDHS 2012-13 and PDHS 2017-18, indicating high reliability. It is pertinent to mention here that data against these variables were not collected during PDHS 2006-07 [35].
  The obstetric characteristics included women’s age at marriage (< 20 years, 20 years and above), women’s age at first birth (< 20 years, 20 years and above), parity (1–2 children, 3–4 children, 5 children or above), number of living children (0, 1–2, 3–4, 5 or above), birth order (1, 2–3, 4–5, 6 or more), antenatal attendance (less than 4 visits or no visit, at least 4 visits or more), sex/gender of the newborn (female, male), and size of the newborn at birth (large, average, small).
2. Community-level characteristics.
  At community level, predictors encompassed regions/provinces (Punjab, Sindh, Baluchistan, Khyber Pakhtunkhwa, Gilgit Baltistan, Islamabad, FATA), respondents’ residential geographical classification (urban, rural), mass media exposure to radio, TV, newspaper (yes, no), and visit of the fieldworker, e.g., Lady Health Worker (LHW) in past 12 months (yes, no). DHS also measured the respondents’ perceived difficulty of distance to the health facility, hindering their access to medical care (problem, not a problem). It is pertinent to mention here that data against respondents’ mass media exposure and perceived difficulty of distance is not available for PDHS 2006-07 [35].
3. Institutional-level characteristics.
  The characteristics at the institutional level consisted of the mode of delivery (vaginal, caesarean section), place of delivery (either at home or public and private hospital/facility), and skilled birth assistance (no, yes). Here, Skilled Birth Assistance (SBA) referred to skilled health professionals, e.g., doctors, nurses, midwives, or Lady Health Visitors (LHVs).

### Data analysis

For data analysis, IBM Statistical Package for Social Sciences (SPSS) version 21 was used and sampling weights were applied. Analysis was conducted at three levels, i.e. univariate, bivariate and multivariate. Firstly, univariate descriptive analysis was performed and presented, using frequencies and percentages. Secondly, cross-tabulation and chi-square test of association (p-value) was conducted, where a p-value ≤ 0.05 was found statistically significant. Lastly, bivariate and multivariate logistic regression was applied to determine the association of maternal and newborn PNC utilization with multi-level determinants at the individual, community, and institutional levels. During bivariate and multivariate regression analysis, odds ratios (ORs) and adjusted odds ratios (AOR) at 95% confidence intervals (CI) were calculated, separately for maternal and newborn PNC utilization. Multivariable logistic regression analysis was carried out after adjusting the visit of fieldworkers (e.g., LHWs) as a constant/fixed variable to obtain the AOR and 95%CI for both maternal and newborn PNC utilization. Further, variance inflation factor (VIF) – a measure to assess multicollinearity was also calculated before multivariate regression, which was reported as > 10 and acceptable [41].

## Results

### Sample characteristics at individual level

Table 1 indicated the individual level (socio-demographic and obstetric) characteristics of MWRAs (15–49 years), who gave birth during 5 years preceding the last three waves of PDHSs from 2006 to 2018. Here, wave 2 represents PDHS 2006-07, wave 3 indicates PDHS 2012-13, and wave 4 highlights PDHS 2017-18.

**Table 1:** Individual level (socio-demographics and obstetric) characteristics of women of reproductive age 15–49 years, who gave a birth during 5 years preceding PDHSs (2006–2018)

Characteristics	PDHS (2006-07)		PDHS (2012-13)		PDHS (2017-18)	
	n = 5,677		n = 7,446		n = 6,711	
	f	%	f	%	f	%
**Individual characteristics**						
**Socio-demographic characteristics**						
**Women’s age**						
15–24 years	1,334	23.5	1,748	23.5	1,545	23
25–34 years	2,952	52	4,038	54.2	3,725	55.5
35 years and above	1,390	24.5	1,659	22.3	1,440	21.5
**Women﻿s’ education status**						
No formal schooling	3,668	64.6	4,155	55.8	3,212	47.9
Up to 5 years of schooling	854	15	1,230	16.5	1,097	16.3
6–10 years of schooling	813	14.3	1,380	18.5	1,492	22.2
More than 10 years of schooling	341	6	682	9.2	911	13.6
**Husbands’ education status**						
No formal schooling	2,007	35.5	2,451	33	1,889	28.7
Up to 5 years of schooling	935	16.5	1,211	16.3	1,085	16.5
6–10 years of schooling	1,904	33.7	2,547	34.3	2,316	35.2
More than 10 years of schooling	812	14.3	1,216	16.4	1,293	19.6
**Women﻿s’ occupation**						
Not working/Unemployed	4,026	71	5,378	72.2	5,528	82.4
Professional/Clerical/Sales & Services	734	12.9	658	8.8	279	4.2
Agriculture	728	12.8	820	11	403	6
Manual or household worker	185	3.3	590	7.9	498	7.4
**Husbands’ occupation**						
Not Working/Unemployed	174	3.1	123	1.7	179	2.7
Professional/Clerical/Sales & Services	1,999	35.2	2,355	31.6	2,119	32.1
Agriculture	1,185	20.9	1,260	16.9	1,154	17.5
Manual or household worker	2,316	40.8	3,707	49.8	3,142	47.7
**Available means of transportation**						
No vehicle	2,523	47.9	3,292	46.7	2,256	34.8
Own vehicle	2,750	52.1	3,761	53.3	4,224	65.2
**Women﻿s’ domestic decision making autonomy** ^**a**^						
No	-	-	1,396	28.3	1,899	34
Yes	-	-	3,545	71.7	3,690	66
**Women﻿s’ emotional autonomy** ^**a**^						
No	-	-	4,175	56.2	3,776	56.3
Yes	-	-	3,251	43.8	2,931	43.7
**Wealth quintile**						
Poorest	1,289	22.7	1,698	22.8	1,444	21.5
Poorer	1,194	21	1,544	20.7	1,299	19.4
Middle	1,099	19.4	1,464	19.7	1,371	20.4
Richer	1,066	18.8	1,469	19.7	1,349	20.1
Richest	1,029	18.1	1,272	17.1	1,248	18.6
**Obstetric characteristics**						
**Women﻿s’ age at marriage**						
< 20 years	4,266	75.1	5,342	71.7	4,322	64.4
20 years and above	1,411	24.9	2,104	28.3	2,389	35.5
**Women﻿s’ age at first birth**						
< 20 years	3,072	54.1	3,685	49.5	3,076	45.8
20 years and above	2,605	45.9	3,761	50.5	3,635	54.2
**Parity**						
1–2 children	2,000	35.2	2,885	38.7	2,749	41
3–4 children	1,648	29	2,249	30.2	2,183	32.5
5 children or above	2,029	35.7	2,312	31.1	1,780	26.5
**Number of living children**						
0	100	1.8	80	1.1	83	1.2
1–2	2,138	37.7	3,149	42.3	2,944	43.9
3–4	1,737	30.6	2,306	31	2,222	33.1
5 or above	1,702	30	1,912	25.7	1,463	21.8
**Antenatal attendance**						
Less than 4 visits or no visit	3,987	71.2	4,713	63.4	2,414	41.1
At least 4 visits or more	1,611	28.8	2,723	36.6	3,452	58.9
**Birth order**						
1	965	17	1,418	19	1,337	19.9
2–3	1,917	33.8	2,710	36.4	2,571	38.3
4–5	1,389	24.5	1,735	23.3	1,739	25.9
6 or more	1,406	24.8	1,583	21.3	1,064	15.9
**Sex/Gender of newborn**						
Female	2,606	45.9	3,583	48.1	3,308	49.3
Male	3,071	54.1	3,863	51.9	3,404	50.7
**Size of newborn at birth**						
Large	1,272	22.6	477	6.4	475	7.1
Average	2,526	44.8	5,421	73	4,862	72.7
Small	1,842	32.4	1,524	20.5	1,353	20.2

^a^ Autonomy related data was not collected during PDHS 2006-07

Table 1 showed that the majority of the MWRAs were aged between 25 and 34 years (52%, 54.2%, and 55.5%) and had not attained formal schooling (64.6%, 55.8%, and 47.9%), however, their husbands had completed 6–10 years of schooling, i.e. 33.7%, 34.3%, and 35.2%. in the last three waves respectively. A common trend was seen in terms of employment during the last three waves of PDHS, where a large number of MWRAs were found unemployed (71%, 72.2%, and 82.4%), while their husbands were mostly employed and working as manual/household workers (40.8%, 49.8%, and 47.7%). Further, mostly MWRAs belonged to the poorest wealth quintile (22.7%, 22.8%, and 21.5%), nonetheless, confirmed the availability of own vehicles for transportation (52.1%, 53.3%, and 65.2%).

With regards to autonomy, relevant data was not collected during PDHS 2006-07. Hence, analysis informed that a substantial proportion of the MWRAs (71.7% and 66%) had domestic decision-making autonomy, whereas 56.2% and 56.3% MWRAs had no emotional autonomy in waves 3 and 4 of PDHS respectively.

Regarding obstetric characteristics of MWRAs in the last three waves, most of the women were married at a younger age, i.e. <20 years (75.1%, 71.7%, and 64.4%). However, a slight variation was observed in women’s age at first birth, as 54.1% of women gave birth at < 20 years in wave 2, while 20 years and above (50.5% and 54.2%) gave birth in wave 3 and 4. More than one-third of MWRAs had 1–2 children ever born (35.2%, 38.7%, and 41%) and similarly 1–2 living children (37.7%, 42.3%, and 43.9%). Further analysis revealed that most of the women had either availed less than 4 antenatal visits or no visit (71.2% and 63.4%) in wave 2 and wave 3 respectively, nonetheless availed at least 4 visits or more (58.9%) in wave 4, showing an increase in antenatal care utilization over time. Furthermore, the majority of MWRAs had 2–3 birth orders (33.8%, 36.4%, and 38.3%), male newborns (54.1%, 51.9%, and 50.7%) with an average size (44.8%, 73%, and 72.7%) in all three waves of PDHS.

### Characteristics at community and institutional level

Table 2 indicated community and institutional level characteristics of MWRAs (aged 15–49 years), who gave birth in the last 5 years preceding PDHSs from 2006 to 2018.

**Table 2:** Community and institutional level characteristics of women of reproductive age 15–49 years, who gave a birth during 5 years preceding PDHSs (2006–2018)

Characteristics	PDHS (2006-07)		PDHS (2012-13)		PDHS (2017-18)	
	n = 5,677		n = 7,446		n = 6,711	
	f	%	f	%	f	%
**Community level characteristics**						
**Regions/Provinces**						
Punjab	3,182	56.1	4,180	56.1	3,453	51.5
Sindh	1,404	24.7	1,714	23	1,571	23.4
Baluchistan	264	4.6	348	4.7	377	5.6
Khyber Pakhtunkhwa^a^	827	14.6	1,117	15	1,101	16.4
Gilgit Baltistan*	-	-	56	0.7	-	-
Islamabad*	-	-	31	0.4	54	0.8
FATA*	-	-	-	-	156	2.3
**Geographical classification**						
Urban	1,714	30.2	2,244	30.1	2,248	33.5
Rural**l**	3,962	69.8	5,202	69.9	4,463	66.5
Mass media exposure*						
No	-	-	2,184	29.4	2,454	36.6
Yes	-	-	5,241	70.6	4,254	63.4
Perceived difficulty of distance to facility*						
Problem	-	-	2,982	40.1	3,024	45.1
Not a problem	-	-	4,451	59.9	3,683	54.9
**Visit of field worker (LHWs)**						
No	4,143	73	2,260	43.2	2,564	38.2
Yes	1,533	27	2,975	56.8	4,147	61.8
**Institutional level characteristics**						
**Mode of delivery**						
Vaginal	5,193	91.5	6,268	84.3	5,094	75.9
C-Section	482	8.5	1,171	15.7	1,614	24.1
**Place of delivery**						
Home	3,545	62.8	3,594	48.3	2,093	31.2
Hospital/Facility (public & private)	2,101	37.2	3,841	51.7	4,618	68.8
**Skilled birth assistance**						
No	3,280	58.1	3,312	44.6	1,879	28
Yes	2,365	41.9	4,112	55.4	4,833	72

^a^ Khyber Pakhtunkhwa was formerly known as North-West Frontier Province (NWFP), as reported in PDHS 2006-07

* Missing information indicates the non-availability of data within PDHS waves, particularly in PDHS 2006-07 for mass media exposure and perceived difficulty of distance to facility

For community-level characteristics, a higher number of women were found from Punjab (56.1%, 56.1%, and 51.5%), and belonged to rural areas (69.8%, 69.9%, and 66.5%). The majority of the MWRAs had exposure to mass media (70.6% and 63.4%) and reported that distance to health facilities was not a problem (59.9% and 54.9%) from wave 3 and 4 respectively. Further, most of the MWRAs (73%) in wave 2 reported that they were not visited by the field workers, e.g., LHWs, nonetheless, an increase in the number of women attended by LHWs (56.8% and 61.8%) was observed in wave 3 and 4.

Regarding institutional level characteristics, a shift from home-based deliveries to hospital-based deliveries was observed from wave 2 to wave 4. Analysis showed that the majority of MWRAs reported that they had a vaginal delivery (91.5%, 84.3%, and 75.9%), however, mostly delivered at home (62.8%), without any SBA (58.1%) in wave 2. Contrary, a higher number of MWRAs were delivered at health facilities/hospitals (51.7% and 68.8%) by SBA (55.4% and 72%) in wave 3 and 4 respectively.

### Maternal and newborn PNC related characteristics

**Fig. 3:**
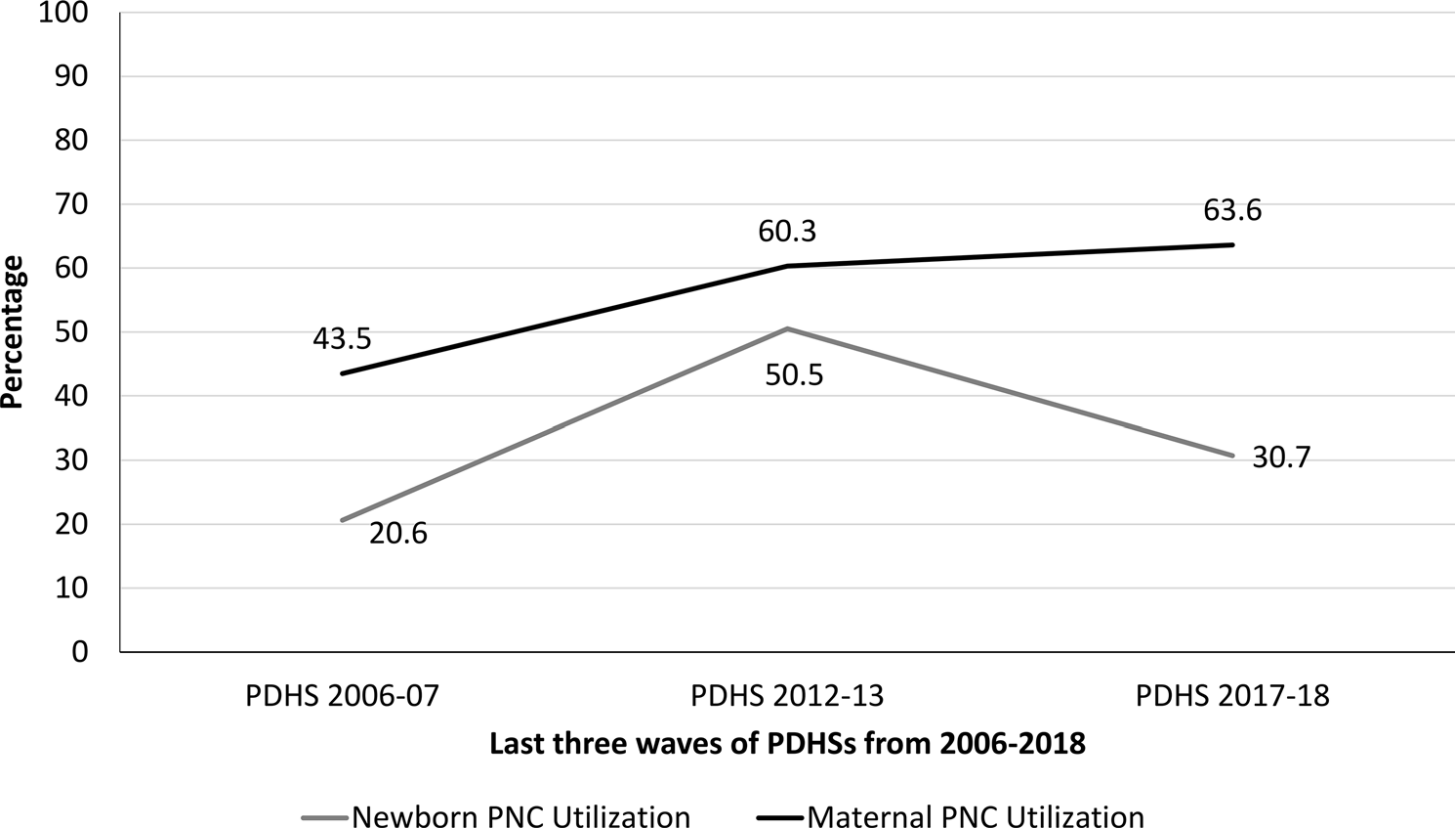
Trends of maternal and newborn PNC utilization in Pakistan from 2006 to 2018

Table 3 highlighted respondents’ characteristics related to maternal and newborn PNC utilization within the first 2 months from 2006 to 2018. Analysis reveals that the trend of maternal and newborn PNC utilization has changed over time from 2006 to 2018, as exhibited in Fig. 3.

**Table 3:** Maternal and newborn postnatal care related characteristics of the women of reproductive age 15–49 years, who gave a birth during 5 years preceding PDHSs (2006–2018)

Characteristics	PDHS (2006-07)		PDHS (2012-13)		PDHS (2017-18)	
	n = 5,677		n = 7,446		n = 6,711	
	f	%	f	%	f	%
**Maternal PNC utilization**						
No	3,181	56.5	2,946	39.7	2,442	36.4
Yes	2,446	43.5	4,473	60.3	4,269	63.6
**Timing for PNC check-up**						
Within first 24 h	2,148	90.1	2,992	81.5	2,510	59.1
Within 2–6 days	177	7.4	391	10.7	117	2.8
Within 7–42 days	59	2.5	289	7.9	1,620	38.2
**Newborn PNC utilization within first 2 months**						
No	2,837	79.4	3,652	49.5	4,631	69.3
Yes	738	20.6	3,724	50.5	2,056	30.7
**PNC utilization by skilled birth attendants**						
No	927	37.9	747	20	400	9.4
Yes	1,518	62.1	2,977	80	3,869	90.6
**Place of 1st PNC check-up**						
Home	980	91.3	800	21.5	508	21
Hospital/Facility (public & private)	94	8.7	2,924	78.5	1,913	79

The majority of respondents informed that they had not availed both maternal PNC (56.5%) and newborn PNC (79.4%) in wave 2, nevertheless, a gradual increase in the proportions of maternal PNC utilization (60.3% and 63.6%) was recorded in wave 3 and 4 respectively, showing an upward trend in maternal PNC utilization. In the case of newborn PNC utilization, a dip was observed, where more than half of the respondents reported that their newborns were checked (50.5%) in wave 3, while 69.3% were not checked after delivery in wave 4, highlighting a downward trend in newborn PNC.

Further, amongst those who availed PNC utilization, the majority availed PNC within the first 24 h (90.1%, 81.5%, and 59.1%), by skilled birth attendants (62.1%, 80%, and 90.6%). Furthermore, a large number of the women reported that their first PNC checkup took place at home (91.3%) in wave 2, nevertheless mostly visited a health facility for their first PNC checkup (78.5% and 79%) during wave 3 and 4 of PDHS.

### Relationship of maternal and newborn PNC utilization with multi-level determinants

Table 4 indicated an association between maternal as well as newborn PNC utilization with individual-level characteristics of MWRAs aged 15–49 years. The results of chi-square showed a statistically significant relationship (p ≤ 0.05) of maternal and newborn PNC utilization with women’s and husband’s education status, women’s occupation, and wealth quintile in the last three waves of PDHS. Further, a strong statistical association between PNC utilization and decision-making, emotional autonomy and available means of transportation was also seen during 3 and 4 waves of PDHS. Furthermore, results of maternal and newborn PNC utilization showed a statistical significance with women’s age and husband’s occupation in varied waves of PDHS.

**Table 4:** Relationship of maternal and newborn PNC utilization with individual level characteristics of women of reproductive age 15–49 years, who gave a birth during 5 years preceding PDHSs (2006–2018)

Characteristics	PDHS (2006-07)						PDHS (2012-13)						PDHS (2017-18)					
	n = 5,677						n = 7,446						n = 6,711					
	Maternal PNC			Newborn PNC			Maternal PNC			Newborn PNC			Maternal PNC			Newborn PNC		
	**Yes**	**No**	**p-value**	**Yes**	**No**	**p-value**	**Yes**	**No**	**p-value**	**Yes**	**No**	**p-value**	**Yes**	**No**	**p-value**	**Yes**	**No**	**p-value**
**Individual characteristics**																		
**Socio-demographic characteristics**																		
**Women﻿s’ age**																		
15–24 years	45.3	54.7	**<0.010**	23.1	76.9	**<0.01**	63.1	36.9	**<0.01**	50.3	49.7	0.79	62.7	37.3	**<0.01**	29.5	70.5	0.13
25–34 years	46.4	53.6		21.8	78.2		60.2	39.8		50.8	49.2		66.1	33.9		31.8	68.2	
35 years and above	35.5	64.5		16.3	83.7		57.5	42.5		49.9	50.1		58.2	41.8		29.5	70.5	
**Women﻿s’ education status**																		
No formal schooling	35.1	64.9	**<0.01**	18.3	81.7	**<0.01**	50.2	49.8	**<0.01**	41.3	58.7	**<0.01**	50.3	49.7	**<0.01**	25.9	74.1	**<0.01**
Up to 5 years of schooling	49.4	50.6		26.1	73.9		60.6	39.4		56.5	43.5		65.7	34.3		33.8	66.2	
6–10 years of schooling	61.6	38.4		31.8	68.2		75.6	24.4		61.1	38.9		76.1	23.9		32.8	67.2	
More than 10 years of schooling	74.5	25.5		29.5	70.5		90.5	9.5		74.4	25.6		87.3	12.7		40.7	59.3	
**Husbands’ education status**																		
No formal schooling	32.5	67.5	**<0.01**	15.7	84.3	**<0.01**	49.6	50.4	**<0.01**	41.3	58.7	**<0.01**	50.7	49.3	**<0.01**	27.5	72.5	**<0.01**
Up to 5 years of schooling	38.9	61.2		20.6	79.4		57.4	42.6		50.3	49.7		57.6	42.4		29.6	70.4	
6–10 years of schooling	49.1	50.9		24.3	75.7		63.6	36.4		52.6	47.4		68.6	31.4		31.8	68.2	
More than 10 years of schooling	62.7	37.3		32.0	68.0		77.6	22.4		64.7	35.3		79.4	20.6		35.1	64.9	
**Women﻿s’ employment/occupation**																		
Not working/Unemployed	44.2	55.8	**<0.01**	20.7	79.3	**<0.01**	62.7	37.3	**<0.01**	51.5	48.5	**<0.01**	64.1	35.9	**<0.01**	29.7	70.3	**<0.01**
Professional/Clerical/Sales & Services	48.4	51.6		23.1	76.9		67.5	32.5		59.0	41.0		75.3	24.7		38.5	61.5	
Agriculture	34.8	65.2		16.3	83.7		45.3	54.7		44.6	55.4		46.9	53.1		30.8	69.2	
Manual or household worker	43.6	56.4		29.8	70.2		51.4	48.6		40.2	59.8		64.8	35.2		37.6	62.4	
**Husbands’ employment/occupation**																		
Not working/Unemployed	38.2	61.8	**<0.01**	14.0	86.0	**<0.01**	50.8	49.2	**<0.01**	38.5	61.5	**<0.01**	59.2	40.8	**<0.01**	34.8	65.2	0.42
Professional/Clerical/Sales & Services	51.6	48.4		23.1	76.9		69.1	30.9		59.3	40.7		72.7	27.3		31.5	68.5	
Agriculture	36.1	63.9		17.0	83.0		54.7	45.3		50.6	49.4		52.9	47.1		31.0	69.0	
Manual or household worker	40.6	59.4		21.4	78.6		56.9	43.1		45.3	54.7		61.7	38.3		30.0	70.0	
**Available means of transportation**																		
No vehicle	39.0	61.0	**<0.01**	20.1	79.9	0.49	52.0	48.0	**<0.01**	43.4	56.6	**<0.01**	56.3	43.7	**<0.01**	28.8	71.2	**0.05**
Own vehicle	46.9	53.1		21.1	78.9		66.9	33.1		56.1	43.9		66.6	33.4		31.2	68.8	
**Women’ decision making autonomy**																		
No	-	-	-	-	-	-	50.9	49.1	**<0.01**	42.6	57.4	**<0.01**	51.3	48.7	**<0.01**	22.1	77.9	**<0.01**
Yes	-	-		-	-		60.3	39.7		51.7	48.3		70.6	29.4		35.2	64.8	
**Women’ emotional autonomy**																		
No	-	-	-	-	-	-	67.3	32.7	**<0.01**	54.6	45.4	**<0.01**	72.0	28.0	**<0.01**	33.3	66.7	**<0.01**
Yes	-	-		-	-		51.3	48.7		45.2	54.8		53.0	47.0		27.4	72.6	
**Wealth quintile**																		
Poorest	31.7	68.3	**<0.01**	17.2	82.8	**<0.01**	43.0	57.0	**<0.01**	36.6	63.4	**<0.01**	47.1	52.9	**<0.01**	27.5	72.5	**<0.01**
Poorer	31.1	68.9		17.9	82.1		50.3	49.7		44.4	55.6		49.7	50.3		27.6	72.4	
Middle	41.0	59.0		20.5	79.5		59.1	40.9		50.3	49.7		63.1	36.9		28.3	71.7	
Richer	52.1	47.9		26.2	73.8		71.7	28.3		58.8	41.2		73.7	26.3		32.0	68.0	
Richest	66.1	33.9		32.9	67.1		83.8	16.2		67.5	32.5		86.9	13.1		39.0	61.0	
**Obstetric characteristics**																		
**Age at marriage**																		
< 20 years	41.2	58.8	**<0.01**	21.5	78.5	**0.01**	55.9	44.1	**<0.01**	46.6	53.4	**<0.01**	57.8	42.2	**<0.01**	28.7	71.3	**<0.01**
20 years and above	50.4	49.6		17.2	82.8		71.5	28.5		60.6	39.4		74.2	25.8		34.5	65.5	
**Age at first birth**																		
< 20 years	40.3	59.7	**<0.01**	21.6	78.4	0.08	54	46	**<0.01**	46	54	**<0.01**	56.4	43.6	**<0.01**	27.9	72.1	**<0.01**
20 years and above	47.2	52.8		19.2	80.8		66.4	33.6		54.9	45.1		69.7	30.3		33.2	66.8	
**Parity (number of children ever born)**																		
1–2 children	49	51	**<0.01**	21.9	78.1	**0.01**	69	31	**<0.01**	55.5	44.5	**<0.01**	70.9	29.1	**<0.01**	34.8	65.2	**<0.01**
3–4 children	45.7	54.3		22.8	77.2		59.4	40.6		49.5	50.5		65.5	34.5		29.3	70.7	
5 children or above	36.2	63.8		18.2	81.8		50.2	49.8		45.2	54.8		50	50		26.2	73.8	
**Number of living children**																		
0	49.5	50.5	**<0.01**	31.5	68.5	**0.01**	75.3	24.7	**<0.01**	53.4	46.6	**<0.01**	66.3	33.7	**<0.01**	29.3	70.7	**<0.01**
1–2	48.3	51.7		22.3	77.7		68.3	31.7		55.5	44.5		70.2	29.8		35	65	
3–4	46	54		21.5	78.5		58.3	41.7		48.4	51.6		64.2	35.8		29.6	70.4	
5 or above	34.5	65.5		17.9	82.1		48.9	51.1		44.7	55.3		49.2	50.8		24	76	
**Antenatal attendance**																		
Less than 4 visits or no visit	33.7	66.3	**<0.01**	18.3	81.7	**<0.01**	48.5	51.5	**<0.01**	43.3	56.7	**<0.01**	54.5	45.5	**<0.01**	28.7	71.3	**<0.01**
At least 4 visits or more	66.9	33.1		34	66		80.8	19.2		63.1	36.9		78.8	21.2		36	64	
**Birth order**																		
1	49.7	50.3	**<0.01**	24.1	75.9	**0.01**	72	28	**<0.01**	57	43	**<0.01**	72.6	27.4	**<0.01**	35.2	64.8	**<0.01**
2–3	47.9	52.1		21.3	78.7		64.3	35.7		52.6	47.4		68.8	31.2		31.8	68.2	
4–5	42.5	57.5		21.9	78.1		54.9	45.1		48.2	51.8		58.7	41.3		28.9	71.1	
6 or more	34.2	65.8		17.3	82.7		48.8	51.2		43.7	56.3		47.9	52.1		25.5	74.5	
**Sex/Gender of baby**																		
Female	42.5	57.5	0.18	18	82	**<0.01**	59.1	40.9	**0.04**	50.3	49.7	0.72	63.3	36.7	0.64	28.8	71.2	**<0.01**
Male	44.3	55.7		22.9	77.1		61.4	38.6		50.7	49.3		63.9	36.1		32.6	67.4	
**Size of baby at birth**																		
Large	46.5	53.5	**0.04**	23.1	76.9	**<0.01**	63.2	36.8	**<0.01**	52.4	47.6	0.17	68.5	31.5	**<0.01**	32.6	67.4	0.30
Average	42.9	57.1		17.6	82.4		62.2	37.8		50.9	49.1		65.3	34.7		30.3	69.7	
Small	42.1	57.9		23.1	76.9		52.5	47.5		48.5	51.5		56.3	43.7		32.1	67.9	

* Chi-square test was applied to determine p-value

Findings highlighted an upward trend of maternal PNC utilization over the period of time, along with a strong association, particularly with women’s older age, higher years of schooling, professional/clerical/sales & services occupation, availability of transportation, and richest wealth quintile, whereas newborn PNC highlighted statistical significance with women’s and their husband’s higher years of schooling across last three waves of PDHS. Results also informed a substantial difference in patterns of newborn PNC utilization with women’s occupations across three waves. Results revealed that a large number of women having decision-making autonomy, nonetheless, without any emotional autonomy received maternal PNC in waves 3 and 4. Contrarily, a considerable variation along with low uptake of newborn PNC utilization was observed with women’s decision-making and emotional autonomy.

With respect to obstetric characteristics, a statistically significant association of maternal and newborn PNC utilization was found with women’s age at marriage, parity, number of living children, antenatal attendance and birth order in the last three waves of PDHS (2006–2018). Nonetheless, a significant relationship of maternal and newborn PNC utilization was also observed with women’s age at first birth, sex/gender and size of the newborn.

Table 5 showed a statistically significant relationship (p ≤ 0.05) between various community-level characteristics with maternal and newborn PNC utilization. Findings informed that majority of the women from Sindh (60.6%, 67.6%, and 75.6%), residing in urban areas (57.8%, 74.4%, and 76.8%) and visited by LHWs (47.5%, 65.2%, and 68.1%) availed maternal PNC utilization. Similar pattern was seen for newborn PNC, where majority of the women living in an urban area (26.4%, 58.3%, and 36.9%), particularly from Sindh province (34.2% and 40.4%) in waves 2 and 4, and Punjab province (58.1%) in wave 3 availed newborn PNC services. Further, those women, who had exposure to mass media and had not faced any difficulty accessing the facility also utilized maternal and newborn PNC during wave 3 and 4 of PDHS. Regarding institutional level characteristics, results highlighted that majority of the women, who had caesarean sections and delivered at health facilities by skilled birth attendants had a strong statistical association with maternal and newborn PNC utilization.

**Table 5:** Relationship of maternal and newborn PNC utilization with community and institutional level characteristics of women of reproductive age 15–49 years, who gave a birth during 5 years preceding PDHSs (2006–2018)

Characteristics	PDHS (2006-07)						PDHS (2012-13)						PDHS (2017-18)					
	n = 5,677						n = 7,446						n = 6,711					
	Maternal PNC			Newborn PNC			Maternal PNC			Newborn PNC			Maternal PNC			Newborn PNC		
	**Yes**	**No**	**p-value**	**Yes**	**No**	**p-value**	**Yes**	**No**	**p-value**	**Yes**	**No**	**p-value**	**Yes**	**No**	**p-value**	**Yes**	**No**	**p-value**
**Community level characteristics**																		
**Regions/Provinces**																		
Punjab	40.3	59.7	**<0.01**	17.5	82.5	**<0.01**	65.5	34.5	**<0.01**	58.1	41.9	**<0.01**	68.5	31.5	**<0.01**	33.3	66.7	**<0.01**
Sindh	60.6	39.4		34.2	65.8		67.6	32.4		50.6	49.4		75.6	24.4		40.4	59.6	
Baluchistan	40.8	59.2		10.7	89.3		39.7	60.3		18.0	82.0		38.7	61.3		15.3	84.7	
Khyber Pakhtunkhwa	27.5	72.5		17.1	82.9		37.4	62.6		33.4	66.6		43.9	56.1		17.4	82.6	
Gilgit Baltistan	-	-		-	-		21.8	78.2		16.1	83.9		-	-		-	-	
Islamabad*	-	-		-	-		80.6	19.4		64.5	35.5		79.2	20.8		38.9	61.1	
FATA*	-	-		-	-		-	-		-	-		30.1	69.9		5.1	94.9	
**Geographical classification**																		
Urban	57.8	42.2	**<0.01**	26.4	73.6	**<0.01**	74.4	25.6	**<0.01**	58.3	41.7	**<0.01**	76.8	23.2	**<0.01**	36.9	63.1	**<0.01**
Rural	37.3	62.7		19.2	80.8		54.2	45.8		47.1	52.9		57.0	43.0		27.6	72.4	
**Access to sources of information**																		
No	-	-	-	-	-	-	42.8	57.2	**<0.01**	37.4	62.6	**<0.01**	48.4	51.6	**<0.01**	22.7	77.3	**<0.01**
Yes	-	-		-	-		67.6	32.4		56.1	43.9		72.4	27.6		35.4	64.6	
**Perceived difficulty of distance to health facility**																		
Problem	-	-	-	-	-	-	49.2	50.8	**<0.01**	40.8	59.2	**<0.01**	54.5	45.5	**<0.01**	26.9	73.1	**<0.01**
Not a problem	-	-		-	-		67.7	32.3		57.0	43.0		71.1	28.9		33.9	66.1	
**Visit of field worker (LHWs)**																		
No	42.0	58.0	**<0.01**	19.6	80.4	**0.01**	62.1	37.9	**0.02**	48.0	52.0	**<0.01**	56.4	43.6	**<0.01**	25.8	74.2	**<0.01**
Yes	47.5	52.5		23.8	76.2		65.2	34.8		58.0	42.0		68.1	31.9		33.8	66.2	
**Institutional level**																		
**Mode of delivery**																		
Vaginal	39.2	60.8	**<0.01**	20.6	79.4	0.44	53.2	46.8	**<0.01**	45.6	54.4	**<0.01**	52.5	47.5	**<0.01**	26.7	73.3	**<0.01**
C-Section	89.0	11.0		33.3	66.7		98.0	2.0		77.0	23.0		98.9	1.1		43.5	56.5	
**Place of delivery**																		
Home	28.7	71.3	**<0.01**	20.3	79.7	**<0.01**	31.6	68.4	**<0.01**	35.2	64.8	**<0.01**	24.7	75.3	**<0.01**	30.7	69.3	0.91
Hospital/Facility (public & private)	68.3	31.7		40.0	60.0		87.1	12.9		65.0	35.0		81.3	18.7		30.8	69.2	
**Skilled birth assistance (type of healthcare provider)**																		
No	28.5	71.5	**<0.01**	19.9	80.1	**<0.01**	30.0	70.0	**<0.01**	33.6	66.4	**<0.01**	23.3	76.7	**<0.01**	29.2	70.8	0.09
Yes	64.2	35.8		27.0	73.0		84.8	15.2		64.3	35.7		79.3	20.7		31.3	68.7	

* Chi-square test was applied to determine p-value

### Bivariate logistic regression of maternal and newborn PNC utilization with multi-level determinants

Table 6 and 4.2 showed the bivariate analysis of maternal and newborn PNC utilization with multi-level determinants at the individual, community, and institutional levels of women of reproductive age 15–49 years, who gave birth during 5 years preceding PDHSs (2006–2018). Overall, the findings of maternal PNC utilization showed that women aged 35 years and above, who attained more than 10 years of schooling, served in professional/clerical/sales & services, and their husbands also had above 10 years of schooling, and were employed as professional/clerical/ sales & services, owned a vehicle for transportation and belonged to the richest wealth quintile were more likely to avail maternal PNC services in all three waves of PDHS. More specifically, maternal PNC utilization increased with the increase in age (OR: 0.66, 0.79, and 0.83), with a higher level of education (OR: 5.39, 9.42, and 6.78), in the category of professional/clerical/sales & services (OR: 1.73, 2.16, and 1.83) and with the increase in wealth quintile (OR: 4.18, 6.86, and 7.42) in all three waves. Additionally, those women, who had decision-making and emotional autonomy had more probability of maternal PNC utilization during waves 3 and 4.

**Table 6:** Bivariate logistic regression of maternal and newborn PNC utilization with individual level characteristics of women of reproductive age 15–49 years, who gave a birth during 5 years preceding PDHSs (2006–2018)

Characteristics	PDHS (2006-07)				PDHS (2012-13)				PDHS (2017-18)			
	n = 5,677				n = 7,446				n = 6,711			
	Maternal PNC		Newborn PNC		Maternal PNC		Newborn PNC		Maternal PNC		Newborn PNC	
	**OR**	**CI (95%)**	**OR**	**CI (95%)**	**OR**	**CI (95%)**	**OR**	**CI (95%)**	**OR**	**CI (95%)**	**OR**	**CI (95%)**
**Individual characteristics**												
**Socio-demographic characteristics**												
**Womens’ age**												
**15–24 years**	**1**		**1**		**1**		**1**		**1**		**1**	
25–34 years	1.04	0.92–1.19	0.93	0.76–1.13	0.88*	0.79–0.99	1.02	0.91–1.14	1.16*	1.03–1.31	1.11	0.97–1.26
35 years and above	0.66*	0.57–0.77	0.65*	0.51–0.82	0.79*	0.69–0.91	0.98	0.86–1.12	0.83*	0.72–0.96	0.99	0.85–1.17
**Womens’ education status**												
**No formal schooling**	**1**		**1**		**1**		**1**		**1**		**1**	
Up to 5 years of schooling	1.80*	1.55–2.09	1.56*	1.25–1.96	1.52*	1.34–1.74	1.85*	1.62–2.10	1.89*	1.64–2.18	1.46*	1.26–1.69
6–10 years of schooling	2.97*	2.53–3.47	2.07*	1.58–2.71	3.06*	2.67–3.52	2.23*	1.97–2.53	3.15*	2.74–3.61	1.39*	1.22–1.59
More than 10 years of schooling	5.39*	4.19–6.95	1.87*	1.07–3.26	9.42*	7.23–12.26	4.13*	3.43–4.97	6.78*	5.51–8.34	1.97*	1.68–2.29
**Husbands’ education status**												
**No formal schooling**	**1**		**1**		**1**		**1**		**1**		**1**	
Up to 5 years of schooling	1.32*	1.12–1.55	1.39*	1.10–1.76	1.37*	1.19–1.57	1.44*	1.25–1.65	1.32*	1.13–1.53	1.11	0.94–1.31
6–10 years of schooling	2.00*	1.76–2.28	1.72*	1.41–2.09	1.77*	1.58–1.98	1.57*	1.41–1.76	2.12*	1.87–2.40	1.23*	1.08–1.41
More than 10 years of schooling	3.50*	2.95–4.15	2.53*	1.92–3.33	3.51*	3.01–4.11	2.61*	2.26–3.01	3.74*	3.18–4.40	1.43*	1.23–1.67
**Womens’ occupation**												
**Not working/Unemployed**	**1**		**1**		**1**		**1**		**1**		**1**	
Professional/Clerical/Sales & Services	1.18*	1.01–1.38	1.15	0.90–1.46	1.24*	1.04–1.47	1.35*	1.14–1.59	1.69*	1.28–2.23	1.47*	1.15–1.89
Agriculture	0.67*	0.57–0.79	0.75*	0.59–0.95	0.49*	0.42–0.57	0.76*	0.66–0.88	0.49*	0.40–0.60	1.05	0.85–1.31
Manual or household worker	0.98	0.72–1.32	1.62*	1.09–2.38	0.63*	0.53–0.75	0.63*	0.53–0.75	1.03	0.85–1.25	1.42*	1.18–1.72
**Husbands’ occupation**												
**Not working/Unemployed**	**1**		**1**		1		**1**		**1**		**1**	
Professional/Clerical/Sales & Services	1.73*	1.25–2.38	1.89*	1.05–3.41	2.16*	1.50–3.11	2.31*	1.59–3.36	1.83*	1.34–2.51	0.86*	0.62–1.19
Agriculture	0.91	0.66–1.27	1.29	0.71–2.35	1.17	0.81–1.69	1.63*	1.11–2.39	0.77	0.56–1.06	0.84*	0.60–1.17
Manual or household worker	1.11	0.81–1.52	1.71	0.95–3.07	1.28	0.89–1.83	1.32	0.91–1.91	1.11	0.82–1.51	0.80*	0.58–1.10
**Available means of transportation**												
**No vehicle**	**1**		**1**		**1**		**1**		**1**		**1**	
Own vehicle	1.38*	1.23–1.54	1.06	0.89–1.25	1.86*	1.69–2.05	1.67*	1.52–1.83	1.55*	1.39–1.72	1.12*	1.00-1.25
**Womens’ decision making autonomy**												
**No**	-	-	-	-	**1**		**1**		**1**		**1**	
Yes	-	-	-	-	1.47*	1.29–1.66	1.44*	1.27–1.63	2.27*	2.03–2.55	1.91*	1.68–2.17
**Womens’ emotional autonomy**												
**No**	-	-	-	-	**1**		**1**		**1**		**1**	
Yes	-	-	-	-	0.51*	0.46–0.56	0.68*	0.62–0.75	0.44*	0.39–0.48	0.75*	0.68–0.84
**Wealth quintile**												
**Poorest**	**1**		**1**		**1**		**1**		**1**		**1**	
Poorer	0.97	0.82–1.15	1.05	0.83–1.32	1.34*	1.17–1.54	1.39*	1.21–1.59	1.11	0.95–1.28	1.01	0.75–1.19
Middle	1.49*	1.26–1.77	1.25	0.98–1.58	1.92*	1.67–2.21	1.75*	1.52–2.02	1.92*	1.65–2.25	1.04	0.88–1.22
Richer	2.33*	1.97–2.76	1.72*	1.34–2.19	3.37*	2.90–3.91	2.47*	2.14–2.86	3.15*	2.69–3.70	1.24*	1.05–1.45
Richest	4.18*	3.51–4.98	2.35*	1.74–3.16	6.86*	5.75–8.19	3.59*	3.08–4.19	7.42*	6.11–9.01	1.68*	1.43–1.97
**Obstetric characteristics**												
**Age at marriage**												
**< 20 years**	**1**		**1**		**1**		**1**		**1**		**1**	
20 years and above	1.45*	1.28–1.64	0.76*	0.61–0.94	1.99*	1.78–2.22	1.76*	1.59–1.96	2.10*	1.89–2.35	1.31*	1.18–1.46
**Age at first birth**												
**< 20 years**	**1**		**1**		**1**		**1**		**1**		**1**	
20 years and above	1.32*	1.18–1.47	0.86	0.73–1.02	1.69*	1.53–1.85	1.43*	1.31–1.57	1.77*	1.60–1.96	1.29*	1.16–1.43
**Parity (number of children ever born)**												
**5 children or above**	**1**		**1**		**1**		**1**		**1**		**1**	
3–4 children	1.48*	1.29–1.69	1.32*	1.08–1.61	1.45*	1.29–1.63	1.19*	1.06–1.34	1.90*	1.67–2.16	1.17*	1.01–1.34
1–2 children	1.69*	1.49–1.92	1.26*	1.03–1.53	2.20*	1.97–2.47	1.52*	1.36–1.69	2.43*	2.15–2.76	1.51*	1.32–1.72
**Number of living children**												
**5 or above**	**1**		**1**		**1**		**1**		**1**		**1**	
3–4	1.62*	1.41–1.86	1.25*	1.02–1.53	1.46*	1.29–1.65	1.16*	1.03–1.31	1.85*	1.62–2.12	1.33*	1.15–1.55
1–2	1.77*	1.56–2.02	1.31*	1.07–1.60	2.25*	2.00-2.53	1.54*	1.37–1.72	2.43*	2.14–2.77	1.71*	1.45–1.97
0	1.86*	1.24–2.80	2.10*	1.16–3.80	3.13*	1.86–5.27	1.40	0.88–2.24	2.02*	1.27–3.23	1.32	0.81–2.15
**Antenatal attendance**												
**Less than 4 visits or no visit**	**1**		**1**		**1**		**1**		**1**		**1**	
At least 4 visits or more	3.98*	3.52–4.50	2.30*	1.88–2.83	4.46	3.99–4.99	2.24*	2.03–2.46	3.11*	2.77–3.48	1.39*	1.25–1.56
**Birth order**												
**6 or more**	**1**		**1**		**1**		**1**		**1**		**1**	
4–5	1.90*	1.61–2.25	1.34*	1.08–1.68	1.28*	1.11–1.46	1.20*	1.05–1.38	1.54*	1.32–1.80	1.18*	0.99–1.40
2–3	1.77*	1.53–2.04	1.30*	1.05–1.61	1.89*	1.67–2.15	1.43*	1.26–1.62	2.40*	2.07–2.78	1.36*	1.16–1.59
1	1.42*	1.22–1.66	1.52*	1.17–1.98	2.71*	2.32–3.15	1.71*	1.47–1.97	2.88*	2.43–3.41	1.58*	1.32–1.89
**Sex/Gender of baby**												
**Female**	**1**		**1**				**1**		**1**		**1**	
Male	1.07	0.97–1.19	1.34*	1.14–1.59	1.10*	1.00-1.21	1.02	0.93–1.11	1.02	0.93–1.13	1.19*	1.07–1.33
**Size of baby at birth**												
**Small**	**1**		**1**		**1**		**1**		**1**		**1**	
Large	1.19*	1.04–1.38	0.99*	0.91–1.23	1.55*	1.26–1.92	1.17	0.95–1.44	1.69*	1.35–2.10	0.89	0.73–1.09
Average	1.03	0.92–1.17	0.71	0.59–0.85	1.49*	1.33–1.67	1.10	0.98–1.23	1.46*	1.29–1.65	0.97	0.78–1.22

* p-value = <0.05

Similar to the above, findings highlighted that probability to avail newborn PNC services augmented with the increase in women age (OR: 0.65), who attained 6–10 years of schooling (OR: 2.07) in wave 2, while more than 10 years of schooling in wave 3 and 4 (OR: 4.13 and 1.97), as well as their husbands also had more than 10 years of schooling (OR: 2.53, 2.61, and 1.43) across three waves of PDHS. Further, the odds of newborn PNC utilization were found higher amongst women serving as professional/clerical/sales & services (OR: 1.35 and 1.47) in waves 3 and 4, and their husbands also working in the same category (OR: 1.89, 2.31, and 0.86) and belonged to richest wealth quintile (OR: 2.35, 3.59, and 1.68) in all three waves of PDHS.

With reference to obstetric characteristics, results also revealed that maternal PNC utilization was found associated with women’s age at marriage and first birth, number of living children, antenatal attendance, birth order, sex/gender of newborn, and size of the newborn at birth. Further data suggested that maternal PNC utilization was higher among women having 1–2 children (OR: 1.69, 2.20, and 2.43) and large size of newborns at birth (OR: 1.19, 1.55, and 1.69) in the last three waves of PDHS. Moreover, results informed that newborn PNC utilization was associated with women age at marriage, the number of living children, antenatal attendance, and birth order. Further data highlighted that odds of newborn PNC utilization were found higher among women having 1–2 children (OR: 1.31, 1.54, and 1.71) in all three waves of PDHS.

Furthermore, Table 7 revealed that women from Sindh were more likely to avail maternal and newborn PNC utilization as compared to other provinces in all three waves. Also, an increased PNC utilization was found in all three waves amongst women residing in urban areas, visited by LHWs, had caesarean section, and delivered at hospital/facility by skilled birth attendants for both mothers and newborn.

**Table 7:** Bivariate logistic regression of maternal and newborn PNC utilization with community and institutional level characteristics of women of reproductive age 15–49 years who gave a birth during 5 years preceding PDHSs (2006–2018)

Characteristics	PDHS (2006-07)				PDHS (2012-13)				PDHS (2017-18)			
	n = 5,677				n = 7,446				n = 6,711			
	Maternal PNC		Newborn PNC		Maternal PNC		Newborn PNC		Maternal PNC		Newborn PNC	
	**OR**	**CI (95%)**	**OR**	**CI (95%)**	**OR**	**CI (95%)**	**OR**	**CI (95%)**	**OR**	**CI (95%)**	**OR**	**CI (95%)**
**Community-level characteristics**												
**Regions/Provinces**												
**Baluchistan**	**1**		**1**		**1**		**1**		**1**		**1**	
Punjab	0.98	0.76–1.27	1.74*	1.12–2.72	2.88*	2.30–3.61	6.29*	4.75–8.34	3.44*	2.77–4.29	2.75*	2.06–3.68
Sindh	2.24*	1.72–2.94	4.28*	2.72–6.76	3.16*	2.49–4.01	4.66*	3.48–6.22	4.91*	3.87–6.22	3.73*	2.77–5.03
Khyber Pakhtunkhwa	0.55*	0.41-0.0.74	1.70*	1.05–2.76	0.91	0.71–1.16	2.28*	1.68–3.08	1.24	0.98–1.58	1.16	0.84–1.59
Gilgit Baltistan	-	-	-	-	0.42*	0.21–0.82	0.83	0.38–1.81	-	-	-	-
Islamabad	-	-	-	-	6.63*	2.62–16.76	8.73*	3.96–19.24	5.88*	2.97–11.66	3.52*	1.90–6.52
FATA	-	-	-	-	-	-	-	-	0.68*	0.45–1.01	0.28*	0.13–0.61
**Geographical classification**												
**Rural**	**1**		**1**		**1**		**1**		**1**		**1**	
Urban	2.30*	2.05–2.59	1.51*	1.25–1.83	2.46*	2.20–2.74	1.57*	1.42–1.74	2.50*	2.23–2.80	1.53*	1.07–1.70
**Access to sources of information**												
**No**	-	-	-	-	**1**		**1**		**1**		**1**	
Yes	-	-	-	-	2.79*	2.52–3.03	2.14*	1.93–2.37	2.79*	2.51–3.09	1.86*	1.65–2.08
**Perceived difficulty of distance to health facility**												
**Problem**	-	-	-	-	**1**		**1**		**1**		**1**	
Not a problem	-	-	-	-	2.16*	1.96–2.37	1.93*	1.75–2.12	2.05*	1.85–2.27	1.39*	1.25–1.54
**Visit of field worker (LHWs)**												
**No**	**1**		**1**		**1**		**1**		**1**		**1**	
Yes	1.25*	1.11–1.41	1.28*	1.07–1.53	1.14*	1.02–1.28	1.49*	1.33–1.66	1.65*	1.45–1.83	1.47*	1.31–1.64
**Institutional-level**												
**Mode of delivery**												
**Vaginal**	**1**		**1**		**1**		**1**		**1**		**1**	
C-Section	12.47*	9.33–16.66	1.71	0.29–10.29	41.51*	27.68–62.25	3.99*	3.45–4.62	85.09*	52.61-137.65	2.12*	1.88–2.38
**Place of delivery**												
**Home**	**1**		**1**		**1**		**1**		**1**		**1**	
Hospital/Facility (public & private)	5.35*	4.76–6.02	2.62*	1.55–4.41	14.66*	13.03–16.49	3.41*	3.10–3.76	13.26*	11.72–15.01	1.01	0.90–1.13
**Skilled birth assistance (type of healthcare provider)**												
**No**	**1**		**1**		**1**		**1**		**1**		**1**	
Yes	4.50*	4.02–5.04	1.48*	1.16–1.90	12.99*	11.61–14.55	3.57*	3.24–3.93	12.61*	11.10-14.33	1.1	0.98–1.24

* p-value = <0.05

### Multivariate logistic regression of maternal PNC utilization with individual, community, and institutional level characteristics

Table 8 exhibited multivariate logistic regression of maternal PNC utilization with individual, community, and institutional level characteristics of women of reproductive age 15–49 years, who gave birth during 5 years preceding PDHSs (2006–2018). While interpreting the results of multivariate logistic regression, one may consider that multivariate analysis was conducted to obtain AOR after controlling for visits of field workers (LHWs). Further, VIF was calculated before multivariate regression to assess multicollinearity, which was found > 10.

**Table 8:** Multivariate logistic regression of maternal PNC utilization with individual, community and institutional level characteristics of women of reproductive age 15–49 years, who gave a birth during 5 years preceding PDHSs (2006–2018)

Characteristics	PDHS (2006-07)			PDHS (2012-13)			PDHS (2017-18)		
	n = 5,677			n = 7,446			n = 6,711		
	AOR	CI (95%)	p-value*	AOR	CI (95%)	p-value*	AOR	CI (95%)	p-value*
**Individual characteristics**									
**Socio-demographic characteristics**									
**Womens’ age**									
**15–24 years**	**1**			**1**			**1**		
25–34 years	0.77	0.51–1.16	0.22	0.85	0.64–1.11	0.23	1.37	0.99–1.89	0.05
35 years and above	0.46*	0.26–0.82	**0.01**	1.25	0.88–1.78	0.20	1.08	0.69–1.69	0.73
**Womens’ education status**									
**No formal schooling**	**1**			**1**			**1**		
Up to 5 years of schooling	1.38*	1.00-1.94	**0.05**	0.70*	0.55–0.88	**<0.01**	0.87	0.66–1.15	0.34
6–10 years of schooling	1.19	0.79–1.79	0.38	0.79	0.59–1.04	0.09	0.91	0.67–1.24	0.55
More than 10 years of schooling	1.53	0.76–3.09	0.23	1.52	0.91–2.53	0.11	0.71	0.45–1.12	0.14
**Husbands’ education status**									
**No formal schooling**	**1**			**1**			**1**		
Up to 5 years of schooling	0.89	0.60–1.30	0.54	1.08	0.86–1.34	0.52	1.04	0.78–1.39	0.77
6–10 years of schooling	0.99	0.71–1.38	0.96	0.98	0.79–1.19	0.81	1.17	0.89–1.53	0.27
More than 10 years of schooling	1.09	0.68–1.75	0.72	1.29	0.95–1.76	0.10	1.19	0.83–1.71	0.35
**Womens’ occupation**									
**Not working/Unemployed**	**1**			**1**			**1**		
Professional/Clerical/Sales & Services	0.92	0.64–1.34	0.67	1.08	0.82–1.42	0.57	0.81	0.51–1.29	0.38
Agriculture	1.21	0.81–1.82	0.35	0.79*	0.62-1.00	**0.05**	0.78	0.51–1.19	0.25
Manual or household worker	0.43	0.18–1.04	0.06	0.76*	0.59–0.98	**0.03**	1.00	0.71–1.41	0.98
**Husbands’ occupation**									
**Not working/Unemployed**	**1**			**1**			**1**		
Professional/Clerical/Sales & Services	1.15	0.50–2.63	0.74	1.22	0.56–2.59	0.61	4.57*	1.23–16.99	**0.02**
Agriculture	0.87	0.37–2.03	0.75	1.12	0.51–2.47	0.78	2.79	0.75–10.40	0.13
Manual or household worker	0.99	0.44–2.25	0.98	1.07	0.49–2.33	0.86	3.95*	1.07–14.57	**0.04**
**Available means of transportation**									
**No vehicle**	**1**			**1**			**1**		
Own vehicle	1.48*	1.13–1.94	**<0.01**	1.14	0.97–1.34	0.12	1.28*	1.03–1.61	**0.03**
**Womens’ decision making autonomy**									
**No**	-			**1**			**1**		
Yes	-	-	-	0.79*	0.66–0.95	**0.01**	1.28*	1.02–1.61	**0.03**
**Womens’ emotional autonomy**									
**No**	-			**1**			**1**		
Yes	-	-	-	1.13	0.96–1.32	0.16	0.77*	0.62–0.95	**0.01**
**Wealth quintile**									
**Poorest**	**1**			**1**			**1**		
Poorer	0.97	0.63–1.49	0.87	1.2	0.96–1.50	0.11	0.74	0.53–1.03	0.07
Middle	1.05	0.67–1.63	0.84	1.02	0.78–1.33	0.91	0.79	0.55–1.15	0.22
Richer	1.17	0.71–1.92	0.54	0.86	0.61–1.19	0.36	0.59	0.38–0.91	0.02
Richest	1.18	0.63–2.18	0.61	1.13	0.73–1.76	0.59	1.69*	1.08–2.92	**0.05**
**Obstetric characteristics**									
**Age at marriage**									
**< 20 years**	**1**			**1**			**1**		
20 years and above	0.69	0.47–1.01	0.06	0.83	0.66–1.06	0.14	1.14	0.86–1.52	0.36
**Age at first birth**									
**< 20 years**	**1**			**1**			**1**		
20 years and above	1.19	0.86–1.67	0.28	1.00	0.82–1.22	0.98	0.96	0.73–1.27	0.78
**Parity**									
**5 children or above**	**1**			**1**			**1**		
3–4 children	0.63	0.34–1.15	0.13	0.86	0.61–1.21	0.39	2.24*	1.39–3.59	**<0.01**
1–2 children	0.57	0.23–1.46	0.24	0.68	0.39–1.18	0.17	4.38*	2.07–9.26	**<0.01**
**Number of living children**									
**5 or above**	**1**			**1**			**1**		
3–4	1.39	0.78–2.46	0.26	1.45*	1.06–1.99	**0.02**	0.69	0.44–1.11	0.13
1–2	1.35	0.58–3.15	0.48	1.94*	1.18–3.19	**0.01**	0.32*	0.16–0.64	**<0.01**
0	2.72	0.65–11.26	0.17	2.37	0.69–8.08	0.17	1.31	0.32–5.35	0.71
**Antenatal attendance**									
**Less than 4 visits or no visit**	**1**			**1**			**1**		
At least 4 visits or more	2.23*	1.63–3.03	**<0.01**	1.69*	1.39–2.05	**<0.01**	1.23*	1.00-1.52	**0.05**
**Birth order**									
**6 or more**	**1**			**1**			**1**		
4–5	0.95	0.59–1.51	0.83	0.99	0.76–1.31	0.99	0.80	0.55–1.18	0.26
2–3	0.88	0.45–1.73	0.72	0.89	0.59–1.33	0.57	0.69	0.41–1.18	0.18
1	0.71	0.31–1.62	0.41	1.24	0.73–2.08	0.42	0.68	0.36–1.29	0.24
**Sex/Gender of newborn**									
**Female**	**1**			**1**			**1**		
Male	0.99	0.77–1.27	0.95	0.97	0.84–1.13	0.99	0.86	0.71–1.05	0.13
**Size of newborn at birth**									
**Small**	**1**			**1**			**1**		
Average	1.03	0.77–1.37	0.85	1.41*	1.17–1.70	**<0.01**	1.69*	1.32–2.16	**<0.01**
Large	1.19	0.86–1.66	0.29	0.92	0.64–1.32	0.66	1.37	0.89–2.11	0.15
**Community-level characteristics**									
**Regions/Provinces**									
**Baluchistan**	**1**			**1**			**1**		
Punjab	0.35*	0.16–0.74	**0.01**	1.45*	1.04–2.04	**0.03**	1.51	0.75–3.03	0.25
Sindh	0.99	0.45–2.15	0.97	1.00	0.71–1.42	0.98	3.24*	1.56–6.74	**<0.01**
Khyber Pakhtunkhwa	0.22*	0.10–0.50	**<0.01**	0.31*	0.21–0.46	**<0.01**	1.3	0.62–2.74	0.49
Gilgit Baltistan	-	-	-	0.08*	0.03–0.25	**<0.01**	-	-	-
Islamabad	-	-	-	0.64	0.17–2.46	0.52	4.44	0.59–33.40	0.15
FATA	-	-	-	-	-	-	0.73	0.11–4.92	0.75
**Geographical classification**									
**Rural**	**1**			**1**			**1**		
Urban	1.34	0.96–1.85	0.08	1.00	0.79–1.26	0.99	0.91	0.70–1.17	0.46
**Mass media exposure**									
**No**	-			**1**			**1**		
Yes	-	-	-	1.10	0.92–1.33	0.29	1.08	0.86–1.37	0.51
**Perceived difficulty of distance to facility**									
**Problem**	-			**1**			**1**		
Not a problem	-	-	-	1.33*	1.12–1.59	**<0.01**	0.87	0.70–1.07	0.18
**Institutional-level characteristics**									
**Mode of delivery**									
**Vaginal**	**1**			**1**			**1**		
C-Section	4.43*	2.32–8.45	**<0.01**	5.33*	3.20–8.89	**<0.01**	24.39*	13.29–44.75	**<0.01**
**Place of delivery**									
**Home**	**1**			**1**			**1**		
Hospital/Facility (public & private)	1.39	0.83–2.37	0.21	3.63*	2.61–5.06	**<0.01**	3.28*	2.17–4.95	**<0.01**
**Skilled birth assistance**									
**No**	**1**			**1**			**1**		
Yes	2.29*	1.38–3.80	**<0.01**	3.22*	2.34–4.42	**<0.01**	1.60*	1.04–2.47	**0.03**

* p-value = <0.05

Multivariable logistic regression analysis was carried out to obtain the AOR after controlling for visit of field worker (LHWs).

VIF was calculated before multivariate regression to assess multicollinearity, which was found < 10

Results (Table 8) informed a significant association of maternal PNC utilization with women’s age of 35 years and above (AOR = 0.46, 95% CI: 0.26–0.82) in wave 2, who attained education up to 5 years of schooling (AOR = 1.38, 95% CI: 1.00-1.94; AOR = 0.70, 95% CI: 0.55–0.88) in waves 2 and 3 respectively, and were employed in agriculture (AOR = 0.79, 95% CI: 0.62-1.00) and manual/household jobs (AOR = 0.76, 95% CI: 0.59–0.98) in wave 3, and their husbands employed as professional/clerical/sales & services (AOR = 4.57, 95% CI: 1.23–16.99) and manual/household jobs (AOR = 3.95, 95% CI: 1.07–14.57) in wave 4. Further, a higher likelihood of maternal PNC was found with availability of own vehicle for transportation (AOR = 0.79, 95% CI: 0.66–0.95; AOR = 1.28, 95% CI: 1.02–1.61) in waves 3 and 4, and richest wealth quintile in wave 4 (AOR = 1.69, 95% CI: 1.08–2.92). Women’s decision-making autonomy (AOR = 0.79, 95% CI: 0.66–0.95; AOR = 1.28, 95% CI: 1.02–1.61) in waves 3 and 4, and emotional autonomy jobs (AOR = 0.77, 95% CI: 0.62–0.95) in wave 4 was also significantly associated with maternal PNC utilization.

Multivariate analysis revealed that maternal PNC utilization showed a strong association with the obstetric characteristics in some waves, such as parity in wave 4, number of living children in waves 3 and 4, and the average size of the newborn at birth in waves 3 and 4. Only the antenatal attendance was found significant with maternal PNC utilization (AOR = 2.23, 95% CI: 1.63–3.03; AOR = 1.69, 95% CI: 1.39–2.05; AOR = 1.23, 95% CI: 1.00-1.52) across all waves of PDHS.

At the community level characteristics, regions/provinces, and perceived difficulty to access the distant health facility highlighted a significant association with maternal PNC utilization during multivariate analysis. More specifically, perceived difficulty was found significant in wave 3, while some of the regions/provinces had a strong association across all waves of PDHS. Further, nearly all characteristics of the institutional level (e.g., mode of delivery, skilled birth assistance) significantly predicted the maternal PNC utilization across waves of PDHS, except for a place of delivery.

### Multivariate logistic regression of newborn PNC utilization with individual, community, and institutional level characteristics

Table 9 informed multivariate logistic regression findings of newborn PNC utilization with individual, community, and institutional level characteristics of women of reproductive age 15–49 years, who gave birth during 5 years preceding PDHSs (2006–2018), after adjusting the visits of field workers (LHWs).

**Table 9:** Multivariate logistic regression of newborn PNC utilization with individual, community and institutional level characteristics of women of reproductive age 15–49 years, who gave a birth during 5 years preceding PDHSs (2006–2018)

Characteristics	PDHS (2006-07)			PDHS (2012-13)			PDHS (2017-18)		
	n = 5,677			n = 7,446			n = 6,711		
	AOR	CI (95%)	p-value*	AOR	CI (95%)	p-value*	AOR	CI (95%)	p-value*
**Individual characteristics**									
**Socio-demographic characteristics**									
**Womens’ age**									
**15–24 years**	**1**			**1**			**1**		
25–34 years	1.71	0.97–2.99	0.06	1.43	0.97–2.09	0.06	1.28	0.99–1.67	0.06
35 years and above	1.35	0.61–2.95	0.45	1.84*	1.14–2.97	**0.01**	1.32	0.92–1.89	0.13
**Womens’ education status**									
**No formal schooling**	**1**			**1**			**1**		
Up to 5 years of schooling	1.22	0.78–1.92	0.38	1.12	0.83–1.52	0.44	0.95	0.75–1.21	0.69
6–10 years of schooling	1.07	0.59–1.94	0.83	1.21	0.85–1.73	0.28	0.76*	0.59–0.99	**0.04**
More than 10 years of schooling	2.17	0.69–6.81	0.18	1.14	0.64–2.01	0.66	0.91	0.62–1.27	0.59
**Husbands’ education status**									
**No formal schooling**	**1**			**1**			**1**		
Up to 5 years of schooling	1.11	0.66–1.88	0.69	1.19	0.89–1.62	0.24	0.97	0.75–1.25	0.81
6–10 years of schooling	1.41	0.89–2.22	0.13	0.86	0.65–1.14	0.30	0.99	0.79–1.27	0.99
More than 10 years of schooling	1.62	0.84–3.11	0.15	1.47	0.97–2.24	0.06	1.32	0.98–1.78	0.06
**Womens’ occupation**									
**Not working/Unemployed**	**1**			**1**			**1**		
Professional/Clerical/Sales & Services	0.89	0.53–1.49	0.66	1.67*	1.19–2.37	**<0.01**	1.08	0.75–1.54	0.68
Agriculture	0.73	0.40–1.30	0.29	0.86	0.61–1.21	0.38	0.77	0.52–1.12	0.17
Manual or household worker	0.81	0.28–2.38	0.71	0.74	0.52–1.05	0.09	1.55*	1.16–2.06	**<0.01**
**Husbands’ occupation**									
**Not working/Unemployed**	**1**			**1**			**1**		
Professional/Clerical/Sales & Services	2.93	0.60-14.25	0.18	0.62	0.11–3.45	0.59	0.55	0.18–1.64	0.28
Agriculture	2.65	0.53–13.36	0.24	0.94	0.17–5.33	0.95	0.70	0.23–2.11	0.53
Manual or household worker	4.09	0.84–1.81	0.08	0.57	0.10–3.16	0.52	0.57	0.19–1.69	0.31
**Available means of transportation**									
**No vehicle**	**1**			**1**			**1**		
Own vehicle	1.25	0.86–1.81	0.24	0.89	0.72–1.12	0.34	0.95	0.78–1.15	0.60
**Womens’ decision making autonomy**									
**No**	-			**1**			**1**		
Yes	-	-	-	0.78	0.59–1.02	0.06	1.56*	1.28–1.90	**<0.01**
**Womens’ emotional autonomy**									
**No**	-			**1**			**1**		
Yes	-	-	-	1.17	0.93–1.47	0.18	1.23*	1.03–1.47	**0.02**
**Wealth quintile**									
**Poorest**	**1**			**1**			**1**		
Poorer	1.24	0.72–2.16	0.44	0.94	0.68–1.29	0.66	0.94	0.70–1.26	0.67
Middle	1.01	0.55–1.84	0.98	0.69*	0.48-1.00	**0.05**	0.83	0.60–1.15	0.27
Richer	1.50	0.78–2.90	0.22	0.75	0.47–1.17	0.20	0.99	0.69–1.44	0.99
Richest	1.44	0.60–3.46	0.41	0.61	0.34–1.07	0.08	1.02	0.66–1.56	0.94
**Obstetric characteristics**									
**Age at marriage**									
**< 20 years**	**1**			**1**			**1**		
20 years and above	0.78	0.43–1.40	0.40	0.89	0.65–1.23	0.49	1.17	0.93–1.47	0.19
**Age at first birth**									
**< 20 years**	**1**			**1**			**1**		
20 years and above	0.55*	0.35–0.88	**0.01**	1.14	0.87-0.1.49	0.35	1.00	0.79–1.27	0.99
**Parity**									
**5 children or above**	**1**			**1**			**1**		
3–4 children	0.98	0.41–2.39	0.97	0.67	0.40–1.12	0.13	0.82	0.44–1.51	0.24
1–2 children	1.44	0.42–4.93	0.56	0.55	0.25–1.23	0.15	0.79	0.53–1.17	0.52
**Number of living children**									
**5 or above**	**1**			**1**			**1**		
3–4	0.72	0.32–1.62	0.42	1.01	0.63–1.61	0.97	1.57*	1.05–2.34	**0.03**
1–2	0.83	0.28–2.48	0.74	1.17	0.58–2.37	0.65	2.06*	1.15–3.67	**0.01**
0	0.55	0.08–3.95	0.55	1.02	0.90–1.98	0.98	2.67*	1.07–7.34	**0.05**
**Antenatal attendance**									
**Less than 4 visits or no visit**	**1**			**1**			**1**		
At least 4 visits or more	1.73*	1.09–2.74	**0.02**	1.09	0.84–1.40	0.51	1.19*	1.09–1.44	**0.05**
**Birth order**									
**6 or more**	**1**			**1**			**1**		
4–5	1.25	0.68–2.29	0.47	1.36	0.93–1.99	0.11	1.13	0.79–1.59	0.49
2–3	1.55	0.63–3.84	0.34	1.64	0.95–2.82	0.07	0.97	0.62–1.52	0.89
1	1.34	0.42–4.26	0.62	1.89	0.91–3.92	0.08	0.99	0.58–1.68	0.97
**Sex/Gender of newborn**									
**Female**	**1**			**1**			**1**		
Male	1.28	0.91–1.80	0.16	1.17	0.96–1.44	0.12	1.28*	1.09–1.49	**<0.01**
**Size of newborn at birth**									
**Small**	**1**			**1**			**1**		
Average	0.77	0.51–1.15	0.19	1.07	0.83–1.39	0.61	0.91	0.74–1.11	0.36
Large	1.04	0.66–1.62	0.87	0.59*	0.38–0.91	**0.01**	0.90	0.64–1.28	0.57
**Community level characteristics**									
**Regions/Provinces**									
**Baluchistan**	**1**			**1**			**1**		
Punjab	1.57	0.48–5.07	0.45	4.28*	1.33–13.77	**0.01**	2.30*	1.09–5.35	**0.05**
Sindh	4.81*	1.47–15.70	**0.01**	5.28*	1.61–17.29	**<0.01**	3.61*	1.53–8.51	**<0.01**
Khyber Pakhtunkhwa	1.93	0.57–6.51	0.29	1.98	0.59–6.64	0.27	1.91	0.79–4.62	0.15
Gilgit Baltistan	-	-	-	0.67	0.12–3.84	0.65	-	-	-
Islamabad	-	-	-	4.79	0.54–42.19	0.16	3.54	0.88–14.31	0.07
FATA	-	-	-	-	-	-	0.30	0.01–19.62	0.57
**Geographical classification**									
**Rural**	**1**			**1**			**1**		
Urban	1.22	0.77–1.92	0.40	0.77	0.57–1.03	0.08	1.09	0.89–1.35	0.36
**Mass media exposure**									
**No**	-			**1**			**1**		
Yes	-	-	-	1.03	0.79–1.34	0.85	1.29*	1.05–1.59	**0.01**
**Perceived difficulty of distance to facility**									
**Problem**	-			**1**			**1**		
Not a problem	-	-	-	1.29*	1.01–1.66	**0.04**	1.11	0.93–1.33	0.24
**Institutional level characteristics**									
**Mode of delivery**									
**Vaginal**	**1**			**1**			**1**		
C-Section	0.70	0.97–1.69	0.79	2.55*	1.71–3.81	**<0.01**	2.23*	1.83–2.72	**<0.01**
**Place of delivery**									
**Home**	**1**			**1**			**1**		
Hospital/Facility (public & private)	0.82	0.16–4.08	0.81	1.26	0.73–2.19	0.40	0.27*	0.18–0.40	**<0.01**
**Skilled birth assistance**									
**No**	**1**			**1**			**1**		
Yes	1.76*	1.09–3.32	**0.05**	2.39*	1.39–4.09	**<0.01**	1.39	0.91–2.14	0.13

* p-value = <0.05

Multivariable logistic regression analysis was carried out to obtain the AOR after controlling for visit of field worker (LHWs)

VIF was calculated before multivariate regression to assess multicollinearity, which was found < 10

Findings of multivariate analysis revealed that those mother, who aged 35 years and above (AOR = 1.84, 95% CI: 1.14–2.97) in wave 3, attained 6–10 years of schooling (AOR = 0.76, 95% CI: 0.59–0.99) in wave 4, serving as professional/clerical/sales & services (AOR = 1.67, 95% CI: 1.19–2.37) in wave 3 and manual/households jobs (AOR = 1.55, 95% CI: 1.16–2.06) in wave 4, belonged to middle wealth quintile (AOR = 0.69, 95% CI: 0.48-1.00) in wave 3 and had both decision-making (AOR = 1.56, 95% CI: 1.28–1.90) and emotional autonomy (AOR = 1.23, 95% CI: 1.03–1.47) in wave 4 are more likely to avail newborn PNC utilization in Pakistan.

Regarding obstetric characteristics, the multivariate analysis highlighted that the mothers gave first birth at the age of 20 years and above (AOR = 0.55, 95% CI: 0.35–0.88) in wave 3, having living children in wave 4, had male newborns (AOR = 1.28, 95% CI: 1.09–1.49) in wave 4 with large size (AOR = 0.59, 95% CI: 0.38–0.91) in wave 3 had more probability to avail newborn PNC services. Findings showed that antenatal attendance with at least 4 visits or more had a strong association with newborn PNC utilization (AOR = 1.73, 95% CI: 1.09–2.74; AOR = 1,19, 95% CI: 1.09–1.44) in waves 2 and 4 respectively.

With reference to community-level characteristics, results showed that women from Sindh province across all three waves of PDHS (AOR 4.81, 95% CI: 1.47–15.70; AOR 5.28, 95% CI: 1.61–17.29; AOR 3.61, 95% CI: 1.53–8.51), while Punjab in last two waves, i.e. wave 3 and 4 (AOR = 4.28, 95% CI: 1.33–13.77) (AOR = 2.30, 95% CI: 1.09–5.35) had higher newborn PNC utilization. Further, those mothers, who had exposure to mass media (AOR = 1.29, 95% CI: 1.05–1.59) in wave 4 and had no perceived difficulty in accessing distant healthcare facilities (AOR = 1.29, 95% CI: 1.01–1.66) in wave 3 are more likely to avail newborn PNC utilization.

Lastly, multivariate analysis with institutional level characteristics informed that newborn PNC utilization had a strong association with mode of delivery (AOR = 2.55, 95% CI: 1.71–3.81; AOR = 2.23, 95% CI:1.83–2.72) in waves 3 and 4, skilled birth assistance (AOR = 1.76, 95% CI: 1.09–3.32; AOR = 2.39, 95% CI: 1.39–4.09) in wave 2 and 3, and place of delivery (AOR = 0.27, 95% CI: 0.18–0.40) in wave 4 of PDHS.

## Discussion

The topic of PNC utilization is of great significance, as it provides a window of opportunity to avert maternal and newborn mortality. The present study is aimed to analyze the trend of multi-level determinants i.e. individual (socio-demographic and obstetric), community, and institutional level determinants, influencing the maternal and newborn PNC utilization among women of reproductive age 15–49 years in Pakistan across the last three waves of PDHS, from 2006 to 2018. This research is an attempt to bridge the gap in existing literature, documenting the maternal and newborn PNC utilization with three hierarchal level determinants at individual, community, and institutional levels to highlight the maternal and newborn health issues in Pakistan.

The research revealed an upward trend in maternal PNC utilization, with an increase from 43.5 to 63.6% from 2006 to 2018 in Pakistan, which highlights the continued efforts of the government at large and the contribution of community-level healthcare providers in particular. Contrary, a dip was observed in newborn PNC utilization in the country, with an upsurge from 20.6 to 50.5% from 2006 to 2013, nonetheless 30.7% in 2018. The decrease in newborn PNC utilization is alarming, that may be attributed to various geographical and health inequalities [42]. Also, it highlights that newborn PNC is the least consulted area due to a lack of awareness amongst mothers [43], and requires immediate attention.

Findings informed that maternal and newborn PNC utilization had a strong association with individual-level socio-demographic characteristics. Particularly, it was found higher amongst older age women, who completed at least basic years of schooling, were employed in various occupations, and had decision-making and emotional autonomy in varied waves of PDHS. These findings related to women’s age, education, and occupation are consistent with the studies conducted in Malawi [44], Nigeria [45], Bangladesh [46, 47], and India [48]. Moreover, the women who had women’s decision-making and emotional autonomy were more likely to utilize PNC services, which also corresponds to the preceding research [44, 49, 50]. A variation between maternal and newborn PNC utilization with some key socio-demographic characteristics was also seen, where the availability of own vehicle for transportation, household wealth quintile, and spouse occupation had a statistically significant relationship with maternal PNC utilization, nonetheless insignificant relationship with newborn PNC utilization. These results are somehow similar to studies carried out in Gondar (Ethiopia), Swaziland (North Africa), and Nepal [50–52]. Arguably, women with improved education, economic status, and empowerment are more capable to seek and afford healthcare services, in contrast to others [53].

A strong association between PNC utilization and women’s obstetric characteristics was observed, particularly with women’s age at first birth, parity, the number of living children, antenatal attendance with at least 4 visits, and sex and size of the newborn. These results are similar to previous studies [54–56], where women who gave birth at age of 25–34 years, had a smaller number of children, with at least 4 antenatal visits, and had male newborns were more likely to avail PNC utilization for themselves and their newborns [57].

Upon analyzing the relationship between newborn PNC utilization and community-level characteristics, the study showed that women living in various regions/provinces of Pakistan (e.g., Sindh & Punjab), who had no perceived difficulty in accessing healthcare facilities to seek medical care were more likely to avail both maternal and newborn PNC utilization. Further, a strong association between exposure to mass media and newborn PNC utilization was also observed, highlighting the significant role of mass media in raising awareness. These findings are also comparable to the previous research, informing that mothers belonged to the developed areas, and had access to mass media as well as health facilities are more advantageous to avail PNC services [58–60].

Regarding institutional level determinants, the study revealed that mode of delivery, skilled birth assistance, and place of delivery in some waves showed a statistically significant relationship with maternal and newborn PNC utilization. These findings informed that births assisted by skilled birth attendants, preferably caesarean section and at health facilities are more likely to receive PNC services. These results are similar to the previous studies [61–67] and also highlight that skilled personnel educate and encourage mothers regarding the significance of PNC. Nonetheless, it is pertinent to mention that PDHS does not collect data on the types of services received during PNC, therefore additional research would be required in this area to develop a more thorough understanding.

### Policy implications

This research suggests multiple policy implications to improve the maternal and newborn PNC utilization in Pakistan. Considering the significance of PNC, this research contributes to expand the policies, particularly ensuring PNC as a critical component of continuum of care, denoting it as a ‘fourth trimester’, instead of a single encounter. This fourth trimester must be indispensable to deliver quality services and support according to the women’ needs, including pre-emptive guidance during pregnancy and development of a postpartum care plan, implementation of comprehensive postpartum visit schedule, and counseling women with pregnancies complicated by preterm birth, gestational diabetes, or hypertensive disorders to control future pregnancies. The results may be helpful for policymakers to include specific implementation strategies directly related to the maternal and newborn survival with defined timelines and releases of appropriate funding for enhancing community access to PNC services. This could be done through establishing and strengthening Emergency Obstetric and Newborn Care (EmONC) centers with equitable geographical distribution and referral support.

Another policy implication highlights to design clearly defined mechanisms and astringent monitoring and evaluation system to gauge effective implementation of strategies and achievement of outcome targets. This could be done through enhancing inter-sectoral collaboration with clearly identified roles and responsibilities of key stakeholders. This research also informs to improve access to health education for girls and women, increasing their social support, and promoting parenting responsibilities for maximizing the benefits of PNC utilization.

## Conclusion

The research concluded that there is a difference in maternal and newborn PNC utilization in Pakistan, which is influenced by multiple individual (socio-demographic and obstetrics), community, and institutional level determinants. Overall, an upward trend in maternal PNC, nonetheless, a downward trend in newborn PNC utilization is evident from the analysis of the last three waves of PDHS from 2006 to 2018, which requires immediate attention. There is a need to promote the benefits of PNC for early diagnosis of postpartum complications, and for saving mothers’ and newborns’ lives.

The research recommends a national-level mass media campaign for health promotion and awareness-raising, preferably in regional languages, and focusing on PNC’s importance, as part of a continuum of care during pregnancy. An active engagement of social media channels can be more beneficial in the present scenario to reach a maximum audience for health promotion. This research also advocates strategizing effective public health interventions to enhance women’s access to healthcare facilities and skilled healthcare providers for childbirth and related PNC services. More specifically, the role of healthcare providers is of utmost importance in health education and counseling for PNC services. Emphasis should be given to educationally and economically marginalized mothers, particularly, those living in remote communities, where PNC utilization is comparatively low.

## Data Availability

The present study used raw data of four waves from PDHS. The DHS Program is authorized to distribute free-of-cost and unrestricted survey data files for academic research with registration only. This dataset is available on the DHS website and may be accessed on the following link https://dhsprogram.com/data/available-datasets.cfm by registering as a DHS user. DHS holds country-specific datasets and is widely used for secondary data analysis across the globe.

## Declarations

The DHS Program maintains ethical standards and ethical reviews to protect the privacy of respondents and household members for all DHS. Furthermore, all questionnaires and procedures for DHS were reviewed and approved by ICF Institutional Review Board (IRB) to comply with the U.S. Department of Health and Human Services regulations for the protection of human subjects (45 CFR 46), as well as by country-specific IRB to ensure compliance of the survey with local laws and norms. You may kindly refer to the following link: https://dhsprogram.com/Methodology/Protecting-the-Privacy-of-DHS-Survey-Respondents.cfm.

All procedures were taken in respect of the Declaration of Helsinki.

## List of abbreviations

AOR: Adjusted odds ratio
CI: Confidence interval
LHW: Lady Health Worker
MMR: Maternal Mortality Ratio
MWRA: Married women of reproductive age
NIPS: National Institute of Population Studies
OR: Odds ratio
PDHS: Pakistan Demographic and Health Survey
PNC: Postnatal care
SBA: Skilled Birth Attendant
SDG: Sustainable Development Goals
SPSS: Statistical Package for Social Sciences
UN: United Nations
VID: Variance inflation factor
